# In-context learning as a retraining-free paradigm for constitutive modeling

**DOI:** 10.1073/pnas.2620983123

**Published:** 2026-10-30

**Authors:** Lingfeng Li, Zhuoyuan Li, Shun Li, Kaixin Zhan, Huajian Gao, Chang Qing Chen, Liu Yang

**Affiliations:** ^a^ Department of Engineering Mechanics, Tsinghua University, Beijing 100084, China; ^b^ Department of Mathematics, National University of Singapore, Singapore 119076, Singapore; ^c^ Institute for Functional Intelligent Materials, National University of Singapore, Singapore 117544, Singapore; ^d^ School of Mathematical Sciences, Peking University, Beijing 100871, China; ^e^ Mechano-X Institute, Tsinghua University, Beijing 100084, China

**Keywords:** constitutive modeling, physics-informed learning, in-context learning, data-driven, solid mechanics

## Abstract

Constitutive modeling is fundamental to computational solid mechanics, yet most existing approaches remain material-specific and require retraining or recalibration when materials change. Here we introduce In-Context Modeling (ICM), a retraining-free paradigm for constitutive modeling that infers constitutive behavior from physical context rather than material-specific optimization. Using hyperelasticity as a testbed, we demonstrate that ICM can be trained once on equilibrium deformation fields spanning diverse materials, geometries, and boundary conditions, enforcing physical equilibrium without stress labels. After training, the same model can be directly deployed on previously unseen hyperelastic materials without retraining. At inference, ICM treats a small set of nonuniform strain fields as physical context to generate constitutive response in a single forward pass. Numerical benchmarks and experimental validation demonstrate accurate stress prediction across out-of-distribution materials and boundary-value problems, together with substantial reductions in deployment cost compared with per-material learning approaches. These results establish in-context learning as a retraining-free paradigm for constitutive modeling, particularly valuable for heterogeneous biological tissues such as the brain, and suggest a pathway toward foundation models for computational mechanics.

## Significance

Constitutive modeling underpins computational mechanics, yet most existing approaches follow a modeling-by-optimization paradigm in which each new material requires calibration or retraining. Here we demonstrate that constitutive modeling can instead be performed through in-context learning. We introduce In-Context Modeling, a physics-informed framework that treats equilibrium deformation fields as physical context and infers constitutive behavior without parameter updates. Using hyperelastic materials as a testbed, we demonstrate retraining-free generalization across previously unseen materials, geometries, and loading conditions, together with finite-element integration and experimental validation. By shifting constitutive modeling from optimization to contextual inference, this work establishes in-context learning as a retraining-free paradigm for constitutive modeling and suggests a pathway toward foundation models in computational mechanics.

Accurate constitutive modeling of hyperelastic materials is a cornerstone of computational mechanics, underpinning simulations in soft robotics (1, 2), biomedical engineering (3, 4), and flexible structures (5, 6). Despite decades of progress, constitutive modeling remains computationally expensive and operationally fragmented: most phenomenological (7–9), data-driven (10–18), and physics-informed approaches (19–26) are inherently material-specific, requiring calibration or retraining when the material changes. This “one model per material” paradigm severely limits scalability, particularly as modern experiments and simulations increasingly generate rich full-field deformation data across diverse material systems (27–29).

A central challenge, therefore, is whether a single constitutive framework can be trained once and then applied directly to previously unseen hyperelastic materials, without retraining or material-specific reformulation. Existing data-driven and physics-informed methods partially address this challenge by reducing reliance on stress measurements through equilibrium or thermodynamic constraints (30–34). From a complementary perspective, recent multitask models for plasticity reduce the material-specific training burden by learning a common core shared across a material family and training only the material-specific layers for a new material, substantially reducing the required data and number of trainable parameters (35). In both cases, however, the material-specific constitutive response remains encoded in network parameters that must be identified for each new material, so deployment costs continue to scale with material diversity.

**Fig. 1.**
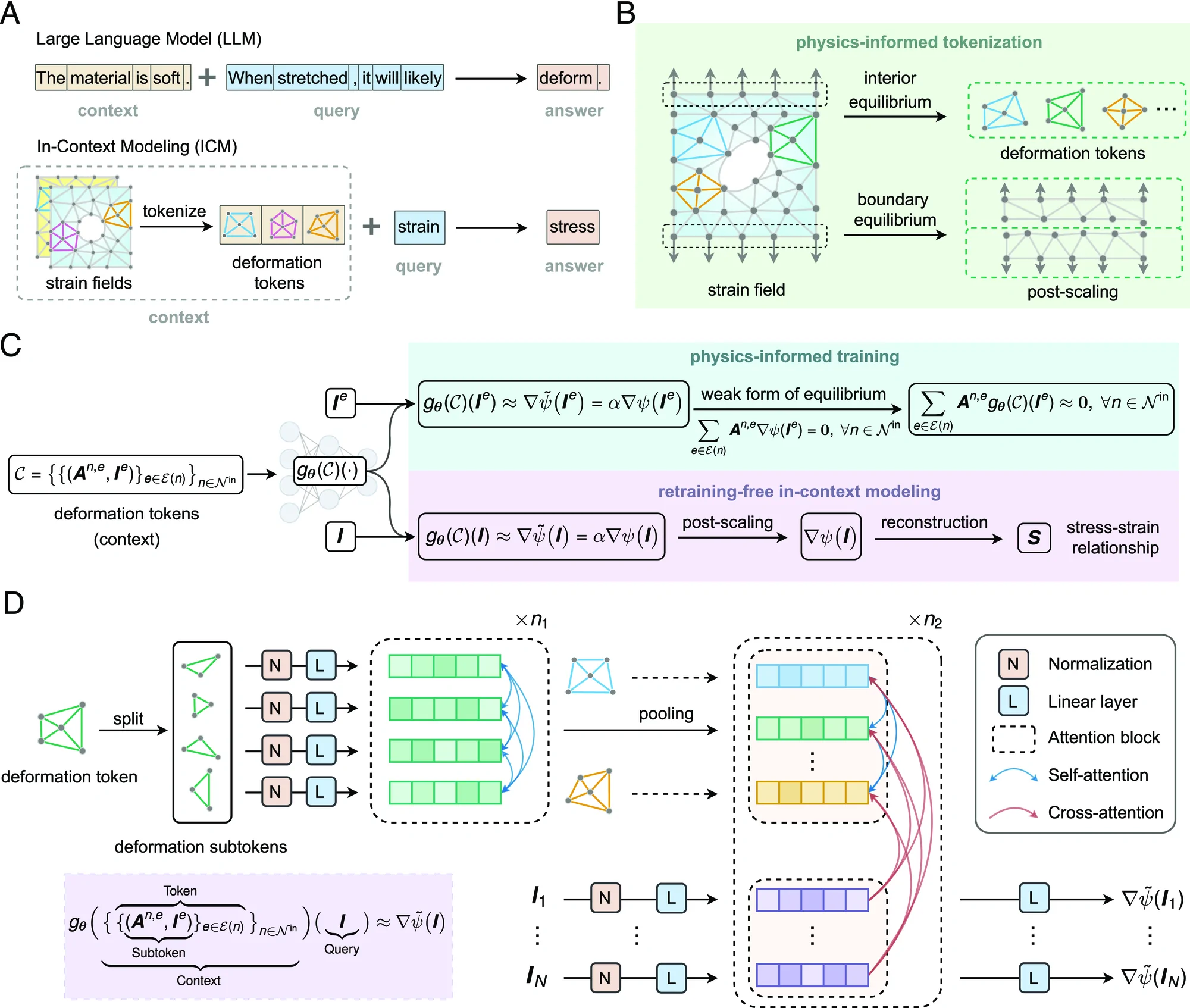
Schematic overview of the ICM paradigm. (*A*) Conceptual analogy between a Large Language Model (LLM) and the proposed In-Context Modeling (ICM) framework. The LLM learns to infer an answer given its textual context; ICM learns to infer the stress–strain relationship given its physical context. (*B*) Physics-informed tokenization in ICM. The strain field is discretized into deformation tokens based on interior equilibrium, while the boundary equilibrium is extracted for the purpose of postscaling. (*C*) The overall workflow of ICM. Assimilating deformation tokens as the physical context, the core network $g_{\theta }$ predicts the scaled strain energy gradient function. In the physics-informed training phase, we enforce interior equilibrium in an unsupervised manner. In the retraining-free in-context modeling phase, we recover the stress–strain relationship via a single forward pass, followed by lightweight postscaling and analytical reconstruction. (*D*) The network architecture of the ICM network $g_{\theta }$. We first map each deformation token into a context embedding by applying normalization, linear projection, self-attention, and average pooling to its subtokens. Concurrently, input strain queries are independently encoded into query embeddings via normalization and linear projection. These context and query embeddings then interact via self- and cross-attention mechanisms to exchange and aggregate information, before a final linear projection which yields the network output.

Here we introduce In-Context Modeling (ICM), which applies in-context learning as a retraining-free paradigm for constitutive modeling, using hyperelasticity as a testbed. The ICM is trained once on equilibrium deformation fields that span a broad range of materials, geometries, and boundary conditions, enforcing physical balance laws without requiring stress labels. After training, the same framework can be directly deployed on previously unseen hyperelastic materials without retraining. The key idea underlying ICM is contextual constitutive modeling: instead of encoding material behavior exclusively in fixed parameters, ICM conditions constitutive response on a small set of measured nonuniform strain fields that serve as physical context. This mirrors the in-context learning paradigm in Large Language Models (LLMs), where a query is answered based on the context (Fig. 1*A*). Given this context, the stress–strain response in queried deformation states is generated in a single forward pass, with absolute stress levels recovered through lightweight calibration using measured external forces. ICM therefore eliminates material-specific retraining, but not the need for material-specific data, which serve as context for inference and calibration rather than as targets for parameter identification or network optimization.

This contextual formulation fundamentally distinguishes ICM from existing physics-informed and equilibrium-based learning approaches. While prior methods reduce data requirements, they remain intrinsically case-specific. ICM instead decouples training from deployment: training extracts shared mechanical structure across a family of hyperelastic responses, whereas deployment relies only on contextual deformation data and corresponding external forces. This separation enables a single trained framework to generalize across materials while maintaining physical consistency, resulting in substantial reductions in deployment cost.

A critical enabler of ICM is the use of spatially nonuniform strain fields as the physical context. Unlike homogeneous tests, nonuniform deformations simultaneously probe a wide range of strain states within a single experiment or simulation, implicitly encoding rich information about material behavior. Such full-field kinematic data are increasingly accessible through modern experimental techniques and high-fidelity simulations (27–29, 36, 37). By leveraging equilibrium constraints, ICM transforms these deformation measurements into constitutive knowledge without pointwise stress data.

In the following, we demonstrate that a single ICM, trained once, accurately predicts stress responses for previously unseen hyperelastic materials across diverse geometries and boundary conditions. Numerical benchmarks and experimental validation highlight both the accuracy and computational efficiency of the framework, establishing ICM as a scalable retraining-free paradigm for constitutive modeling in computational mechanics. By amortizing constitutive learning across materials, ICM transforms constitutive modeling from a per-material optimization task into a reusable computational platform.

## Results

### In-Context Modeling.

Given a set of observed equilibrium strain fields and boundary conditions for an unknown isotropic hyperelastic material, we aim to infer the stress–strain relationship (12, 16).

We begin with the weak formulation of force equilibrium in the absence of body forces (38, 39):

[1] $$\begin{array}{c} \int_{\Omega }P:\nabla v\;\;\text{d}V=0,\;\forall v\in V, \end{array}$$

where Ω denotes the domain, P is the first Piola–Kirchhoff stress tensor, and v is the test function in the space V.

By applying discretization and tensor transformations (*Derivation of Modeling Objective in Hyperelasticity*), Eq. **1** yields

[2] $$\begin{array}{c} \sum_{e\in E(n)}A^{n,e}\nabla \psi \left(I^{e}\right)=0,\;\forall n\in N^{\mathrm{in}}, \end{array}$$

where $N^{\mathrm{in}}$ represents the aggregated set of all interior nodes collected from strain fields, E(n) denotes the set of elements connected to node n, $I^{e}$ is the strain invariants of element e, ψ is the strain energy density function, and $A^{n,e}$ is the coefficient matrix associated with n and e, determined by the local deformation and mesh configuration. For isotropic hyperelastic materials, ψ is a function of I, and we use ∇ψ to denote its gradient with respect to input I. Physically, each equation establishes the nodal force equilibrium for an interior node, with the summation terms accounting for the contributions from adjacent elements.

Based on Eq. **2**, we can aggregate the set ${\left\{\left(A^{n,e},I^{e}\right)\right\}}_{e\in E(n)}$ into a “deformation token,” where each individual term $\left(A^{n,e},I^{e}\right)$ acts as a “deformation subtoken” (Fig. 1*B*). Such physics-informed tokenization translates the governing equilibrium into a structured representation. By encapsulating the local equilibrium surrounding an interior node and stripping away ad hoc global domain geometry and loading conditions, each token acts as the atomic unit of information regarding the stress–strain relationship. This inherent modularity allows us to freely sample and aggregate tokens from an arbitrary number of strain fields (*Network Inputs and Architecture*). When these tokens are assembled to form the physical context $C={\left\{{\left\{\left(A^{n,e},I^{e}\right)\right\}}_{e\in E(n)}\right\}}_{n\in N^{\mathrm{in}}}$, they provide a comprehensive representation of the interior equilibrium across strain fields.

The core of ICM is a neural network $g_{\theta }$ with trainable parameters θ, which approximates ∇ψ given the context C, up to a scaling factor (Fig. 1 *C* and *D*). By mapping the physical context directly to the scaled energy density gradient instead of specific material parameters, ICM is inherently agnostic to the mathematical form of the strain energy, naturally generalizing to unseen material classes. Formally, the network treats the deformation tokens as the physical context, takes the strain invariants I as the query, and predicts the corresponding scaled energy gradient at the queried I, i.e.,

[3] $$\begin{array}{l} g_{\theta }\underset{\text{Context}}{\underset{︸}{\left({\left\{\overset{\text{Token}}{\overset{︷}{{\left\{\underset{\text{Subtoken}}{\underset{︸}{\left(A^{n,e},I^{e}\right)}}\right\}}_{e\in E(n)}}}\right\}}_{n\in N^{\text{in}}}\right)}}\;\left(\underset{\text{Query}}{\underset{︸}{I}}\right)\\ \;\;\;\;\;\;\approx \nabla \tilde{\psi }(I)=\alpha \nabla \psi (I), \end{array}$$

where α is the scaling factor that can be calculated during inference. To address the spatial heterogeneity in mesh resolution and the variation in strain ranges, we normalize all deformation tokens and queries utilizing the scale invariance of Eq. **2**. This preprocessing step ensures that network inputs remain invariant to nonphysical discretization artifacts and are adaptively scaled to the contextual deformation state. For the network architecture, the ICM network adopts self- and cross-attention mechanisms (40), which not only generalize to a variable number of deformation tokens and subtokens, but also maintain their permutation invariance, respecting the unordered nature of the nodal equilibrium equations (*Network Inputs and Architecture*).

By substituting the neural approximation Eq. **3** into the discretized equilibrium equations Eq. **2**, we obtain

[4] $$\begin{array}{c} \sum_{e\in E(n)}A^{n,e}g_{\theta }\left(C\right)\left(I^{e}\right)\approx 0,\;\forall n\in N^{\mathrm{in}}. \end{array}$$

The ICM network is trained by minimizing the residuals of Eq. **4** as a physics-informed loss that enforces discretized equilibrium. This formulation enables unsupervised learning without the need for ground-truth stress information.

As Eq. **2** is invariant to the scaling of the network prediction, an additional postscaling process is required to determine the scaling factor α during inference. To this end, we revisit the weak form of equilibrium Eq. **1** on the boundaries, which implies

[5] $$\begin{array}{c} \sum_{n\in N_{j,k}^{b}}\sum_{e\in E(n)}A^{n,e}\nabla \psi \left(I^{e}\right)=F_{j,k},\;\forall \left(j,k\right)\in B, \end{array}$$

where $N_{j,k}^{b}$ indicates all boundary nodes under the j-th boundary condition of the k-th contextual strain field, $F_{j,k}$ is the corresponding external force, and B denotes the index set of all (j,k) pairs. To obtain the scaling factor α, we can compute $\alpha_{j,k}$ for each (j,k) pair by comparing the true external force magnitude with the inferred one calculated by replacing ∇ψ with $g_{\theta }$ in Eq. **5**, and then average $\alpha_{j,k}$ over B as the estimated α. Once ∇ψ is obtained, the stress S is analytically reconstructible. We refer to this rescaling as the postscaling procedure (*Training and Inference*). This scale-agnostic training and postscaling approach is critical to the model’s generalization across materials: ICM decouples the profile of the stress–strain relationship from its absolute magnitude, allowing the network to remain robust even for materials exhibiting orders-of-magnitude variations in stress.

### Dataset and Evaluation Setup.

**Fig. 2.**
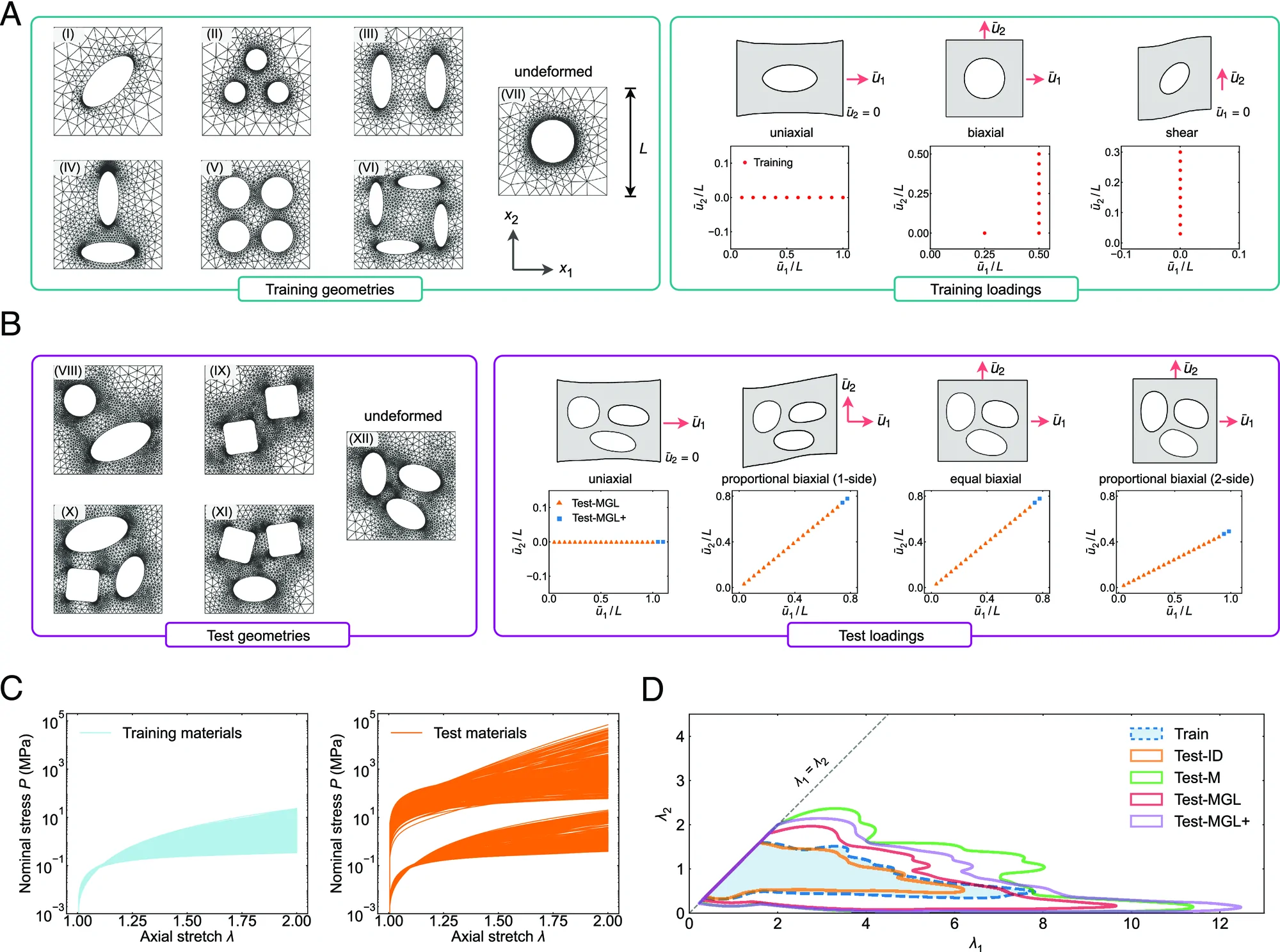
Configurations of the training and test sets. (*A*) Geometries and loading modes in the training dataset, including 7 plate geometries with different hole shapes and arrangements (labeled I-VII), 3 loading modes, and 10 loading steps for each loading mode. (*B*) Geometries (labeled VIII–XII) and loading modes in the Test-MGL and Test-MGL+ sets. Note the inclusion of unseen square-hole geometries and new loading modes not present in (*A*). (*C*) Material differences between training (*Left*) and test (*Right*) sets, shown by uniaxial nominal stress-stretch (P-λ) curves, highlighting orders-of-magnitude variations in stress levels. (*D*) Distribution of deformation states in training and test sets, shown in the principal stretch space ($\lambda_{1}$-$\lambda_{2}$). The contours represent the effective coverage of each dataset, showing that the test sets span regions extending beyond the training domain, thereby providing a comprehensive testbed for evaluating the model’s out-of-distribution generalization.

For a training dataset covering diverse scenarios, we generated 2,000 hyperelastic material models defined by polynomial strain energy functions (*SI Appendix*, *Dataset*) and combined them with seven plate geometries with different numbers and arrangements of circular or elliptical holes (Fig. 2*A*). Each geometry is subjected to uniaxial tension, biaxial tension, and in-plane shear, producing complex, nonuniform strain fields. Altogether, the dataset contains over 500 million (M) deformation tokens and captures a broad spectrum of hyperelastic mechanical responses.

To verify robust generalization, we construct four test sets that progressively increase the data diversity: i) Test-ID evaluates in-distribution prediction by replacing the training materials with 400 unseen polynomial hyperelastic models while keeping the training geometries and loading modes unchanged. ii) Test-M introduces 500 unseen materials drawn from four other common strain energy forms, namely Ogden (8, 41), Pucci-Saccomandi (PS) (42), Exp-Ln (43), and van der Waals (VdW) (44, 45) (*SI Appendix*, *Dataset*), again under the training geometries and loading modes. iii) Test-MGL uses the same materials as Test-M, but associates them with five unseen geometries and additional loading modes (Fig. 2*B*). iv) Test-MGL+ further increases the maximum loading magnitudes in Test-MGL by 10%. This incremental setup provides a multifaceted lens for examining how the model generalizes across increasingly complex unseen scenarios, spanning from new materials to unexplored geometric and loading configurations.

Material differences between training and test sets are summarized in Fig. 2*C*, highlighting the orders-of-magnitude variations in stress levels between them. Fig. 2*D* compares the distribution of deformation states in each dataset. Notably, the test sets span regions extending beyond the training domain, reflecting the compounded effects of distribution shifts in materials, geometries, and loadings, thereby providing a comprehensive testbed for evaluating the model’s out-of-distribution generalization.

### Generalization in Materials, Geometries, and Loadings.

With strategic designs in tokenization, preprocessing and network architecture, as well as the training scheme, we expect to equip ICM with a generalizable capability to infer intrinsic stress–strain relationships from physical context. To validate this expectation, we train an ICM network with 4 M parameters for $3.4\times 10^{5}$ steps, and evaluate the relative prediction error ${\left(\cdot \right)}^{\text{err}}$ (*Training and Inference*), treating each individual tokenized deformation field from test sets as both context and query.

**Fig. 3.**
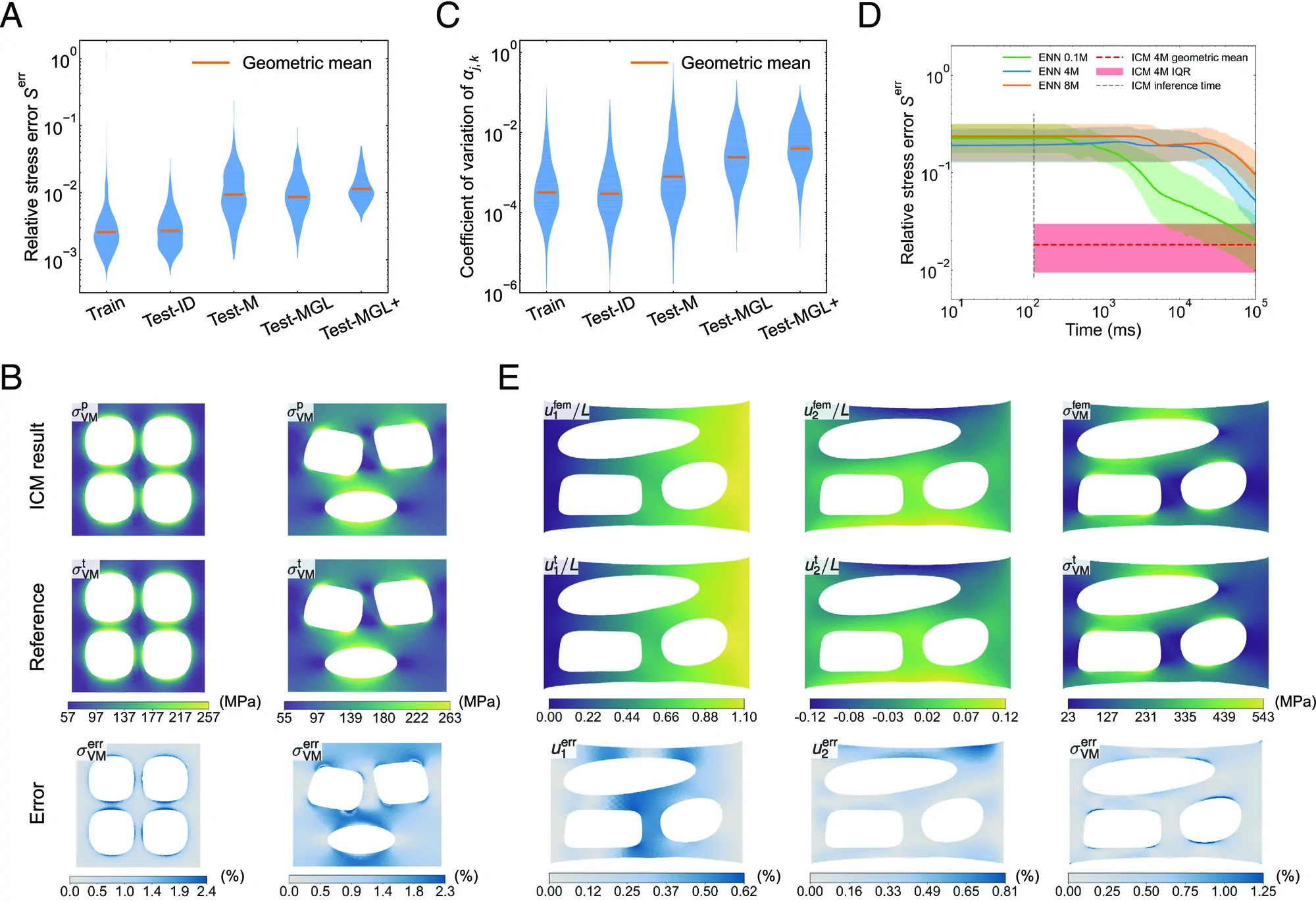
Generalization performance and finite-element validation of ICM. (*A*) Relative stress error $S^{\text{err}}$ on the training set and the four test sets, where the blue shaded areas and orange lines denote the underlying distributions and geometric means across samples, respectively. The relative errors remain in the order of $10^{-2}$ for all datasets, showing robust generalization. (*B*) Visualization of von Mises stress fields for two representative samples from Test-M (*Left*) and Test-MGL+ (*Right*). Each column shows the prediction ($\sigma_{\text{VM}}^{\text{p}}$), ground truth reference ($\sigma_{\text{VM}}^{\text{t}}$), and spatial distribution of relative error ($\sigma_{\text{VM}}^{\text{err}}$). (*C*) Coefficients of variation for the scaling factors $\alpha_{j,k}$ on the training set and the four test sets, confirming consistency among different boundary conditions. (*D*) Comparison between ICM with 4 M parameters and the Equilibrium-based Neural Network (ENN) with 0.1 M, 4 M, and 8 M parameters. ICM achieves lower errors while reducing computation time by approximately three orders of magnitude. The solid lines and shaded regions represent the geometric mean and interquartile range (IQR) across test materials, respectively. (*E*) ICM-driven finite-element simulations in FEniCS for a Test-MGL+ sample under uniaxial displacement loading (${\overset{¯}{u}}_{1}/L=1.1$). Predicted displacement components ($u_{1}^{\text{fem}}/L$ and $u_{2}^{\text{fem}}/L$, visualized with a scale factor of 0.5), and von Mises stress ($\sigma_{\text{VM}}^{\text{fem}}$) closely match the reference finite-element solution.

Fig. 3*A* summarizes the results across the training and test sets, with representative examples visualized in Fig. 3*B*. Even for the most challenging Test-MGL+ dataset, the relative errors remain in the order of $10^{-2}$, showing robust generalization. Interestingly, the prediction error for Test-MGL is slightly lower than that for Test-M. This counterintuitive result is, in fact, a direct manifestation of our physics-informed design. As revealed in Fig. 2*D*, despite the introduction of unseen geometries and loading modes, the local deformation states in Test-MGL are actually closer to the training domain than those in Test-M. Since our method relies exclusively on these local states via the tokenization strategy, the lower prediction error in Test-MGL naturally follows.

For the postscaling step, the final scaling factor is obtained by averaging the individual factors $\alpha_{j,k}$ derived from each boundary condition. The minimal coefficients of variation (SD divided by the mean) observed across all datasets in Fig. 3*C* confirm strong agreement among the factors inferred from different boundary conditions.

Existing equilibrium-based unsupervised stress inference methods are typically trained separately for each material without cross-material generalization (31–34). We compared ICM with the Equilibrium-based Neural Network (ENN) (32), a representative baseline for unsupervised stress inference (*SI Appendix*, *Comparison with the ENN Model*). This comparison is appropriate because both methods are trained using only the force equilibrium and do not require stress labels. For each test material, the same ten deformation fields from Test-M were used to train the material-specific ENN and as context for the pretrained ICM, and both methods were evaluated on the same Test-MGL+ fields. We used a 4 M-parameter ICM and ENNs with 0.1 M, 4 M, and 8 M parameters. The deployment time includes material-specific training and query prediction for ENN, but only context-conditioned inference for ICM because no parameter update is required. As shown in Fig. 3*D*, ICM achieves lower relative stress errors while reducing the overall deployment time by approximately three orders of magnitude compared with all ENN variants.

To demonstrate practical applicability, we integrated the trained ICM network as a constitutive model into the open-source finite-element software FEniCS (46, 47) (Fig. 3*E*). In this pipeline, ICM first infers the material’s stress–strain relationship from a provided context of 10 deformation fields, which is then utilized in FEniCS to solve a displacement loading problem. Notably, the geometry and loading configurations in the FEM simulation are entirely distinct from those in the ICM input context. The resulting displacement and von Mises stress fields show excellent agreement with ground truth, confirming that the stress–strain relationship inferred by ICM is fully compatible with conventional FEM pipelines.

### Experimental Validation and Test-Time Scaling Behavior.

To test whether ICM can infer accurate stress–strain relationships from experimentally measurable quantities, specifically the full-field strain measurements from Digital Image Correlation (DIC) (27–29, 36, 37, 48) and the external forces recorded by the testing machine, we validated ICM via real-world experiments on two hyperelastic materials, classified as “soft” and “hard” based on their relative stiffness (*SI Appendix*, *Experimental Setup*).

**Fig. 4.**
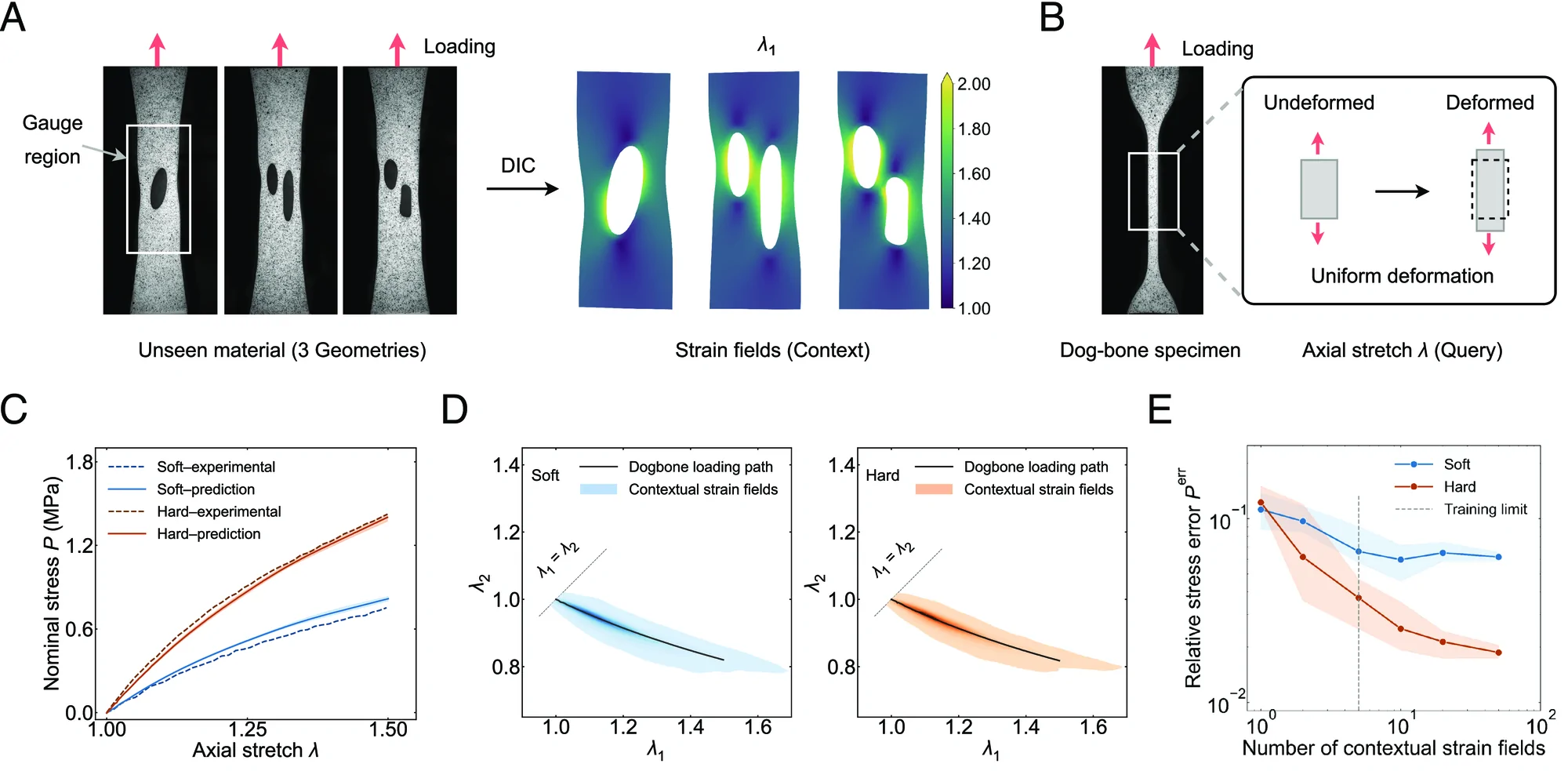
Experimental validation of ICM. (*A*) Nonuniform strain fields were captured by applying DIC to 3D-printed perforated specimens under uniaxial tension. These fields, visualized by the maximum principal stretch $\lambda_{1}$, serve as the physical context for the network after tokenization. (*B*) The uniform deformation, induced by independent uniaxial tests on standard dog-bone specimens and measured via DIC, is extracted as the query state. (*C*) Comparison between the ICM-inferred nominal stress-stretch (P-λ) curves and the experimental ground truth. The predictions show excellent agreement for both materials without any material-specific retraining. (*D*) Deformation state distributions in the principal stretch ($\lambda_{1}$-$\lambda_{2}$) space. The nonuniform contextual strain fields (shading indicates data density) provide broad coverage that fully envelops the 1D strain trajectory of the dog-bone query, driving the accurate stress predictions in (*C*). (*E*) Test-time scaling and context length extrapolation. The geometric mean (solid lines) and interquartile range (IQR) (shaded regions) of the relative nominal stress error $P^{\text{err}}$ across random context samplings both decay with richer contextual strain fields. Notably, the sustained error reduction far beyond the training context length limit (vertical dashed line) demonstrates a remarkable capability for context length extrapolation at inference.

For each material, 3D-printed perforated specimens with distinct geometries were tested under uniaxial tension to induce nonuniform deformation fields (Fig. 4*A*), which were recorded via DIC to serve as context inputs for ICM. Given the absence of ground-truth stress–strain relationships, direct stress comparisons are infeasible. To establish a quantitative comparison, a standard dog-bone specimen of the same material was independently tested under uniaxial tension (Fig. 4*B*). This setup yielded a uniform deformation field measured by DIC, alongside nominal stress derived directly from the external force. By utilizing tokenized nonuniform fields as context and the uniform dog-bone strain as the query, the ICM-predicted stresses exhibit excellent agreement with the experimental ground truth for both materials (Fig. 4*C*). To explain this performance, Fig. 4*D* visualizes the contextual deformation states, which broadly cover the entire strain trajectory of the dog-bone query, thereby enabling the highly accurate stress predictions.

In modern Large Language Models, the emerging paradigm of “test-time scaling” demonstrates that a model’s performance can consistently improve by allocating additional resources during inference, such as processing richer contextual information. ICM exhibits an analogous behavior. As shown in Fig. 4*E*, both the prediction error and its interquartile range decrease with more context. Notably, this scaling behavior shows a remarkable capacity for context length extrapolation. While the network was trained with a limited range of up to 1,500 tokens sampled from a maximum of five strain fields, it successfully extrapolates to an order of $5\times 10^{4}$ tokens from 50 strain fields during inference with continual performance gains.

### Learning the Latent Manifold.

**Fig. 5.**
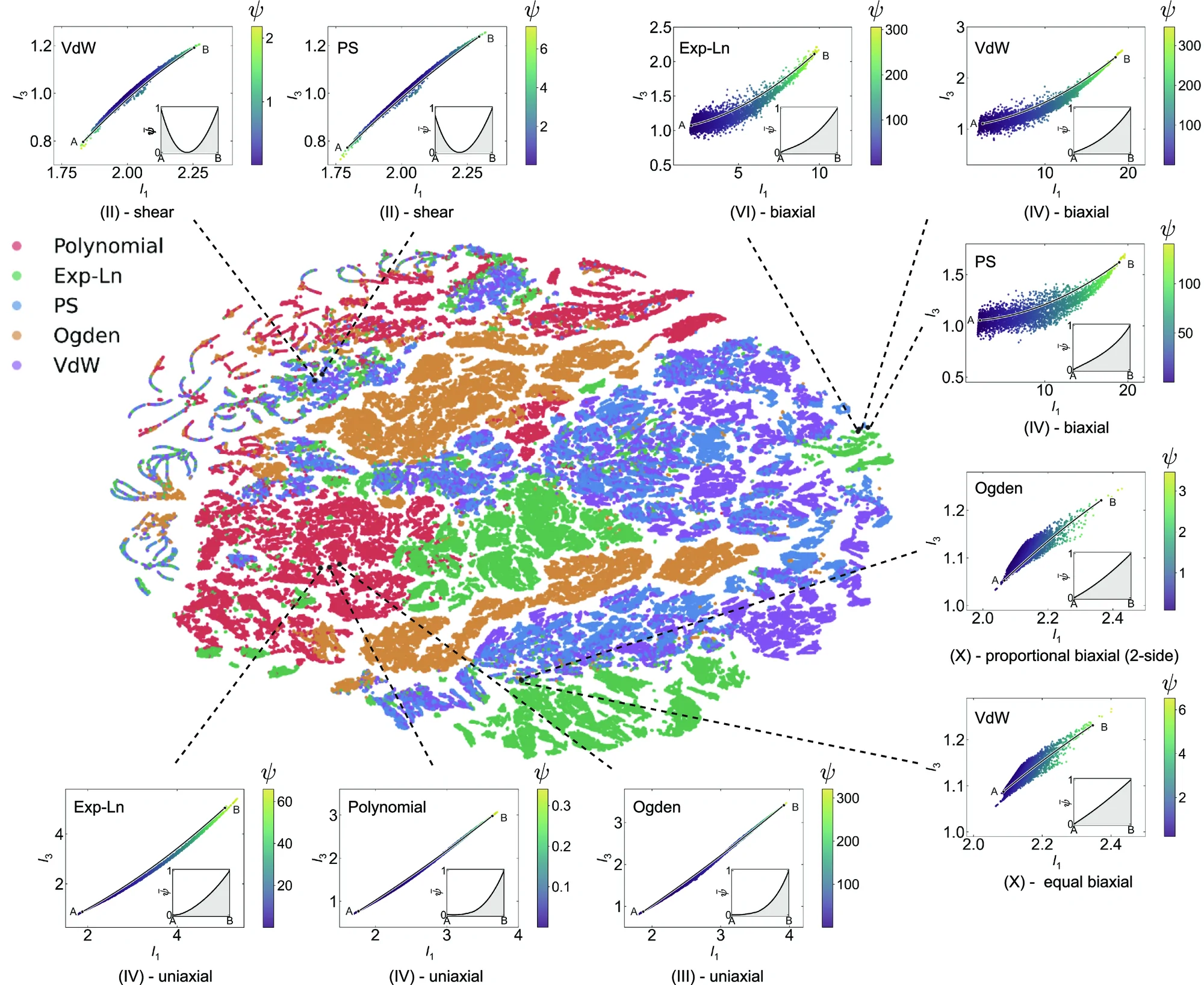
Visualization of the learned context embedding manifold. We projected the average-pooled context embeddings of diverse deformation fields into a two-dimensional t-SNE space. The *Insets* visualize the underlying strain energy density ψ restricted to $\left(I_{1},I_{3}\right)$ states within each deformation field, labeled with their material classes, geometries, and loading modes. In each *Inset*, a curve from A to B marks the selected deformation path, and the smaller panel shows the corresponding normalized energy $\overset{¯}{\psi }$ along the path. Nearby points in the t-SNE visualization often exhibit similar normalized strain-energy profiles across different material classes, energy magnitudes, geometries, and loading conditions. A quantitative local-neighborhood analysis performed directly in the original embedding space further supports the association between embedding proximity and scale-normalized constitutive-response similarity (*SI Appendix*, *Quantitative Analysis of Context Embeddings*).

Having demonstrated the robust empirical generalization of ICM across unseen testing scenarios, we now investigate the driving mechanisms. To this end, we analyzed the model’s latent representations across all test sets, encompassing five material classes, 12 geometries, and seven loading modes. Specifically, we visualized the average-pooled final-layer context embeddings of each deformation field using the t-distributed Stochastic Neighbor Embedding (t-SNE) technique (49) (Fig. 5). The t-SNE projection provides a qualitative visualization of how ICM organizes diverse physical scenarios within its latent space.

The visualization reveals that adjacent points within the t-SNE manifold correspond to deformation fields whose strain energy density functions share nearly identical profiles, regardless of the underlying material classification or the absolute magnitude of the strain energy. Furthermore, context representations derived from deformation fields with different geometries and loading modes can also converge to adjacent latent regions, provided their energy density functions share similar profiles. We further quantified this trend through a local-neighborhood analysis performed directly in the original embedding space (*SI Appendix*, *Quantitative Analysis of Context Embeddings*).

These observations suggest that ICM fulfills our design expectations: by distilling vast datasets, it learns a latent manifold whose local organization reflects similarities in stress–strain relationship profiles across different material classes and strain energy magnitudes. By projecting complex physical context onto this latent manifold, ICM realizes a shared mechanism of in-context inference, thereby driving the robust generalization previously demonstrated.

### Train-Time Scaling Behavior.

**Fig. 6.**
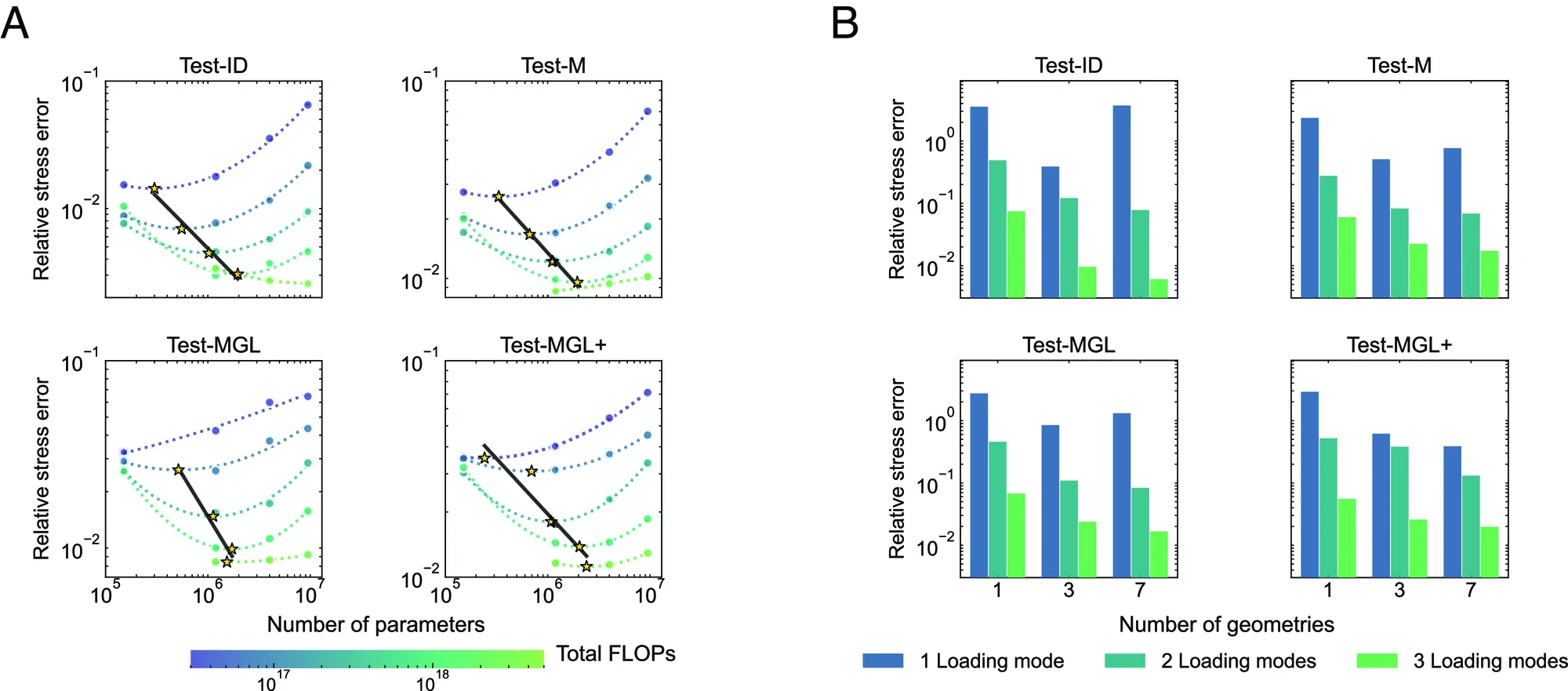
Train-time scaling behavior of ICM over computational budget and data diversity. (*A*) Relative stress error as a function of model size (number of parameters) and computational budget across the four test sets. Each point represents a network configuration, colored by total floating-point operations (FLOPs). Dashed curves fit configurations sharing identical FLOPs via quadratic regression, and yellow stars mark the optimal model size that minimizes the error for each computational budget. The black lines fit these optima, illustrating that coupling greater computational budgets with appropriately larger architectures consistently drives down prediction errors. (*B*) Relative stress error evaluated as a function of training-data diversity on the same test sets. The horizontal axis denotes the number of training geometries (1 for (VII) only, 3 for (II)/(V)/(VII), and 7 for all training geometries), while the color indicates the number of loading modes (1, 2, or 3) included in the training set, with results for 1- and 2-mode configurations averaged across all possible combinatorial selections. The monotonic decay in error confirms that the model benefits from expanding the diversity of the training set.

Having demonstrated the remarkable test-time scaling capabilities of ICM, we now turn to its train-time scaling behavior. In particular, we study the reduction in prediction error along two orthogonal dimensions: i) training computational budget and ii) training data diversity (Fig. 6). To rigorously isolate these effects, we performed two complementary studies: one scaling model size and computational budget with a fixed dataset, and the other one scaling data diversity while fixing the model size and computational budget.

For the first study, we use the same training dataset as other sections, and train a family of ICM networks across a wide spectrum of parameter counts and training steps, quantifying the computational budget via total floating-point operations (FLOPs) (Fig. 6*A*). We estimated the minimum testing error across various model sizes for a given computational budget, which yields an empirical scaling behavior: an increase in the computational budget drives a consistent reduction in prediction error, provided the model size is appropriately scaled up.

For the second study, we fix the 4 M model size and $1.0\times 10^{5}$ training steps, and vary the diversity of the training set along two axes: the number of geometries and loading modes. As shown in Fig. 6*B*, the relative stress error decays as data diversity increases. This shows ICM’s remarkable capacity to harness data diversity, effectively translating rich training scenarios into performance gains.

Collectively, these results confirm that, at least within the scope of our experiments, ICM consistently benefits from increased training resources. By successfully leveraging broader data diversity and greater computational budgets to achieve lower prediction errors, the model demonstrates a scalable behavior that holds great promise for training future physical foundation models.

## Discussion

This work introduces In-Context Modeling (ICM) as a retraining-free constitutive framework for hyperelastic materials. By training once on equilibrium deformation fields spanning diverse materials, geometries, and loading conditions, ICM can be directly applied to previously unseen hyperelastic materials without material-specific retraining. This capability addresses a central scalability limitation in computational mechanics, where constitutive modeling workflows typically rely on repeated calibration or optimization for each new material system.

The central conceptual advance of ICM is its contextual formulation of constitutive modeling. Rather than encoding material behavior entirely in fixed model parameters, ICM conditions constitutive response on a small set of nonuniform deformation fields that serve as physical context. These contextual strain fields implicitly probe a wide range of deformation states and, when combined with equilibrium constraints, provide sufficient information to infer material response without stress labels. This shifts constitutive modeling from per-material parameter identification to context-conditioned response generation.

ICM is fundamentally distinct from existing physics-informed and equilibrium-based learning approaches, which, despite reducing reliance on stress data, remain intrinsically case-specific and require retraining for each new material. ICM instead decouples training from deployment. During training, it extracts shared mechanical structure across a broad family of hyperelastic responses. During deployment, the fixed network uses material-specific nonuniform deformation fields as physical context and measured external forces for postscaling, without any parameter updates. This separation substantially reduces deployment cost while maintaining accuracy under pronounced distribution shifts. Its practical advantage therefore lies in the direct use of these data without pointwise stress labels, repeated model selection, or material-specific optimization. The ability to infer constitutive behavior from physical context without material-specific retraining could be particularly valuable for biological tissues such as the brain, where mechanical properties are heterogeneous and vary across anatomical regions, individuals, and physiological or pathological states.

The generalization capability of ICM stems from its ability to leverage shared mechanical response patterns across materials. Hyperelastic materials governed by different strain-energy formulations may exhibit similar normalized stress–strain behavior over relevant deformation regimes. The learned contextual representations reflect these shared response patterns, as supported by the quantitative neighborhood analysis of the embedding space. Together with the separation of response structure and magnitude, this provides a plausible basis for ICM’s observed retraining-free generalization across materials with widely varying stiffness and nonlinearity.

The experimental validation further demonstrates that constitutive response patterns learned from synthetic training data can transfer to experimental measurements from two real materials over the deformation range examined. However, the constitutive response space represented during training is still defined by prescribed analytical models. A promising direction is to augment synthetic pretraining with a large-scale experimental database comprising full-field deformation measurements and external forces from diverse materials and loading conditions, which could progressively expand the learned response space.

The present study focuses on quasi-static, isotropic hyperelasticity, primarily in two dimensions. Extending ICM to three dimensions would require volumetric deformation fields, with experimental data potentially obtained using techniques such as digital volume correlation (DVC) (36, 37). Extension to anisotropic materials represents a separate challenge: the current isotropic invariant representation would need to incorporate information about material directions, for example through structural tensors and the corresponding anisotropic invariants. History dependence and inelastic behavior would further require time-ordered representations of both the physical context and query, together with a mechanism for representing the evolving internal states. The equilibrium-based training principle would remain applicable at each loading increment, but sufficiently diverse loading histories would be required to learn the corresponding path-dependent responses. We leave these extensions to future work.

More broadly, the underlying formulation is not limited to constitutive modeling: analogous affine constraints can also arise in other physical modeling problems, such as nonlinear diffusion, after suitable discretization and reformulation (*Derivation of Modeling Objective in Nonlinear Diffusion*). These observations suggest that the underlying in-context formulation may extend beyond constitutive modeling to other physical problems governed by analogous constraints. Exploring such extensions represents an important direction for future work.

In conclusion, the In-Context Modeling establishes a retraining-free constitutive framework for hyperelastic materials. By leveraging equilibrium deformation fields as physical context, ICM enables a single trained model to generalize across previously unseen materials without retraining. The demonstrated accuracy, computational efficiency, and experimental compatibility highlight contextual, physics-informed learning as a promising route toward scalable and general-purpose constitutive modeling in computational mechanics.

## Materials and Methods

### Derivation of Modeling Objective in Hyperelasticity.

In this section, we show how the equilibrium equations are discretized and reformulated into the physics-informed tokenization.

Let $X\in \Omega \subset R^{2}$ denote the material coordinates in the reference configuration and u(X) the displacement field. The deformation gradient is $F=I+\nabla_{X}u$, where I is the identity tensor. For isotropic materials, the strain energy density ψ depends on the invariants of the right Cauchy–Green tensor $C=F^{⊤}F$. In two dimensions, we consider

[6] $$\begin{array}{c} I_{1}=\;\text{tr}\;C,\;I_{3}=\;\det C. \end{array}$$

Here, $I_{2}$ is omitted because it is fully determined by $I_{1}$ and $I_{3}$. We therefore denote the invariant vector by $I=(I_{1},I_{3})$ with the index set defined as M={1,3}.

For a hyperelastic material, the second Piola–Kirchhoff stress S is given by

[7] $$\begin{array}{c} S_{\mathrm{lr}}=2\;\frac{\partial \psi }{\partial C_{\mathrm{lr}}}=2\;\frac{\partial I_{m}}{\partial C_{\mathrm{lr}}}\;\frac{\partial \psi }{\partial I_{m}}. \end{array}$$

This relation shows that the stress can be expressed in terms of the gradient of the strain energy density with respect to the invariants.

To derive the discretization, we consider the weak formulation of the force equilibrium equations in the absence of body forces (38, 39), given by

[8] $$\begin{array}{c} \int_{\Omega }P:\nabla v\;\;\text{d}V-\int_{\partial \Omega_{t}}\overset{¯}{t}\cdot v\;\;\text{d}S=0,\;\forall v\in V, \end{array}$$

where Ω denotes the reference configuration of the domain with boundary decomposition $\partial \Omega =\partial \Omega_{u}\cup \partial \Omega_{t}$, and P is the first Piola–Kirchhoff stress tensor. The functional space V comprises sufficiently smooth test functions satisfying v=0 on the Dirichlet boundary $\partial \Omega_{u}$. Here, $\overset{¯}{t}$ represents the traction on the Neumann boundary $\partial \Omega_{t}$. We assume displacement-controlled loading such that $\overset{¯}{t}=0$ on $\partial \Omega_{t}$, thus the second integral term vanishes.

Using the shape functions $N^{n}$ associated with node n, we approximate the displacement field via linear interpolation

[9] $$\begin{array}{c} u\left(X\right)=\sum_{n}N^{n}\left(X\right)\;u^{n}, \end{array}$$

where $u^{n}\in R^{2}$ is the nodal displacement. Substituting this approximation into the weak form (Eq. **8**) and choosing admissible test functions v yield the nodal force components

[10] $$\begin{array}{c} f_{i}^{n}=\int_{\Omega }P_{\mathrm{ir}}\;\frac{\partial N^{n}}{\partial X_{r}}\left(X\right)\;\;\text{d}V\;\left(i=1,2\right), \end{array}$$

where Einstein summation notation is adopted here and throughout the subsequent derivations. The discretized equilibrium equations can then be written as

[11] $$\begin{array}{c} \begin{array}{cccc} f^{n} & =0, & \forall n\in N^{\mathrm{in}}, & \text{(interior equilibrium)}\\ \sum_{n\in N_{j,k}^{b}}f^{n} & =F_{j,k}, & \forall (j,k)\in B, & \text{(boundary equilibrium)} \end{array} \end{array}$$

where $f^{n}=\left(f_{1}^{n},f_{2}^{n}\right)$ is the nodal force, $N^{\mathrm{in}}$ is the set of interior nodes, $N_{j,k}^{b}$ is the boundary node set for the j-th boundary condition in the k-th strain field, $F_{j,k}$ is the corresponding external force, and B denotes the index set of all (j,k) pairs.

Substituting P=FS and Eq. **7** into Eq. **10** yields

[12] $$\begin{array}{c} f_{i}^{n}=\int_{\Omega }2\;F_{\mathrm{il}}\;\frac{\partial I_{m}}{\partial C_{\mathrm{lr}}}\;\frac{\partial \psi }{\partial I_{m}}\;\frac{\partial N^{n}}{\partial X_{r}}\;\;\text{d}V, \end{array}$$

which is linear with respect to $\partial \psi /\partial I_{m}$.

We mesh Ω using $N_{\text{e}}$ linear triangular elements. For linear triangles, $\nabla_{X}N^{n}$ and F are constant within each element, so the integral in Eq. **12** can be evaluated exactly by element-wise quadrature

[13] $$\begin{array}{c} f_{i}^{n}=\sum_{e=1}^{N_{\text{e}}}w^{e}P_{\mathrm{ir}}^{e}\frac{\partial N^{n}}{\partial X_{r}^{e}}=\sum_{e=1}^{N_{\text{e}}}2w^{e}F_{\mathrm{il}}^{e}\frac{\partial I_{m}^{e}}{\partial C_{\mathrm{lr}}^{e}}\frac{\partial \psi }{\partial I_{m}^{e}}\frac{\partial N^{n}}{\partial X_{r}^{e}}, \end{array}$$

where the superscript e denotes the element index and $w^{e}$ is the reference area of element e. We aggregate the coefficients of $\partial \psi /\partial I_{m}^{e}$ as

[14] $$\begin{array}{c} A_{\mathrm{im}}^{n,e}:=2w^{e}F_{\mathrm{il}}^{e}\frac{\partial I_{m}^{e}}{\partial C_{\mathrm{lr}}^{e}}\frac{\partial N^{n}}{\partial X_{r}^{e}}, \end{array}$$

and consequently the discrete nodal force can be written compactly as

[15] $$\begin{array}{c} f_{i}^{n}=\sum_{e=1}^{N_{\text{e}}}A_{\mathrm{im}}^{n,e}\frac{\partial \psi }{\partial I_{m}^{e}},\;i\in \left\{1,2\right\}. \end{array}$$

Since $\partial N^{n}/\partial X_{r}^{e}\neq 0$ only for the elements adjacent to node n, we may equivalently restrict the sum to the neighboring elements as

[16] $$\begin{array}{c} f_{i}^{n}=\sum_{e\in E(n)}A_{\mathrm{im}}^{n,e}\frac{\partial \psi }{\partial I_{m}^{e}},\;i\in \left\{1,2\right\}, \end{array}$$

where E(n) is the set of elements connected to node n.

Combining Eq. **16** with Eq. **11** and denoting the matrix form of $A_{\mathrm{im}}^{n,e}$ as $A^{n,e}$ and the vector form of $I_{m}^{e}$ as $I^{e}$, we recover the discrete equilibrium equations in the form of Eqs. **2** and **5**

[17] $$\begin{array}{c} \begin{array}{cc} \sum_{e\in E(n)}A^{n,e}\nabla \psi \left(I^{e}\right) & =0,\;\forall n\in N^{\mathrm{in}},\\ \sum_{n\in N_{j,k}^{b}}\sum_{e\in E(n)}A^{n,e}\nabla \psi \left(I^{e}\right) & =F_{j,k},\;\forall \left(j,k\right)\in B. \end{array} \end{array}$$

### Derivation of Modeling Objective in Nonlinear Diffusion.

In this section, we present nonlinear diffusion as another example of the ICM paradigm, illustrating how the same methodology extends beyond hyperelasticity to a different physical setting through an appropriate physics-informed tokenization.

Let $x\in \Omega \subset R^{2}$ denote the spatial coordinate and c(x,t) the concentration field. We consider a diffusion process governed by Fick’s law (50)

[18] $$\begin{array}{c} q=-D(c)\nabla c, \end{array}$$

where q is the diffusive flux and $D\left(c\right)\in R^{2\times 2}$ is the diffusivity tensor. In two dimensions, we consider

[19] $$\begin{array}{c} D\left(c\right)=\left[\begin{array}{cc} D_{11}\left(c\right) & D_{12}\left(c\right)\\ D_{12}\left(c\right) & D_{22}\left(c\right) \end{array}\right]. \end{array}$$

To derive the discretized transport equations, we consider the weak formulation of the mass conservation equation in the absence of volumetric source terms, formulated as

[20] $$\begin{array}{c} \int_{\Omega }v\;\frac{\partial c}{\partial t}\;\;\text{d}V+\int_{\Omega }\nabla v\cdot (D\left(c\right)\nabla c)\;\;\text{d}V=0,\;\forall v\in V, \end{array}$$

where Ω denotes the spatial domain, and the functional space V comprises sufficiently smooth scalar test functions satisfying v=0 on ∂Ω, since Dirichlet boundary conditions are imposed on the entire boundary.

Using the shape functions $N^{n}$ associated with node n, we approximate the concentration field at time $t^{m}$ by

[21] $$\begin{array}{c} c^{m}\left(x\right)=\sum_{n}N^{n}\left(x\right)\;c^{n,m}, \end{array}$$

where $c^{n,m}$ denotes the nodal concentration at time $t^{m}$, and we write $c^{m}\left(x\right):=c\left(x,t^{m}\right)$. For each time step m, substituting the finite-element approximation into Eq. **20** and applying a backward-Euler discretization in time yield

[22] $$\begin{array}{c} \int_{\Omega }N^{n}\frac{c^{m}-c^{m-1}}{\Delta t^{m}}\;\;\text{d}V+\int_{\Omega }\nabla N^{n}\cdot (D\left(c^{m}\right)\nabla c^{m})\;\;\text{d}V=0,\;\forall n\in N^{\mathrm{in}}, \end{array}$$

where $N^{\mathrm{in}}$ denotes the set of interior nodes.

We mesh Ω using $N_{\text{e}}$ linear triangular elements. For each element e, both $\nabla N^{n}$ and $\nabla c^{m}$ are constant. We further approximate the diffusivity tensor as constant within each element and evaluate it at the representative element concentration

[23] $$\begin{array}{c} c^{e,m}:=\frac{1}{|\Omega^{e}|}\int_{\Omega^{e}}c^{m}\left(x\right)\;\;\text{d}V. \end{array}$$

For linear triangular elements, $c^{e,m}$ is equivalently the concentration at the element centroid. The nodal balance Eq. **22** can thus be written as

[24] $$\begin{array}{c} \sum_{e=1}^{N_{\text{e}}}A_{\mathrm{ij}}^{n,e,m}D_{\mathrm{ij}}\left(c^{e,m}\right)=\sum_{e=1}^{N_{\text{e}}}b^{n,e,m}, \end{array}$$

where

[25] $$\begin{array}{c} A_{\mathrm{ij}}^{n,e,m}:=w^{e}\frac{\partial N^{n}}{\partial x_{i}^{e}}\frac{\partial c^{m}}{\partial x_{j}^{e}}, \end{array}$$

and

[26] $$\begin{array}{c} b^{n,e,m}:=-\int_{\Omega^{e}}N^{n}\frac{c^{m}-c^{m-1}}{\Delta t^{m}}\;\;\text{d}V. \end{array}$$

Since $N^{n}$ vanishes on elements not adjacent to node n, we may equivalently restrict the summation to the neighboring elements as

[27] $$\begin{array}{c} \sum_{e\in E(n)}A^{n,e,m}:D\left(c^{e,m}\right)=b^{n,m},\;\forall n\in N^{\mathrm{in}},\;\forall m\geq 1, \end{array}$$

where E(n) is the set of elements connected to node n, $A^{n,e,m}$ denotes the matrix form of $A_{\mathrm{ij}}^{n,e,m}$, and $b^{n,m}=\sum_{e\in E(n)}b^{n,e,m}$. This yields a local affine constraint on the constitutive response D(c), which serves as the modeling objective in nonlinear diffusion.

### Network Inputs and Architecture.

The core of the ICM workflow is a network $g_{\theta }$ defined in Eq. **3**. Given a set of deformation tokens ${\left\{\left(A^{n,e},I^{e}\right)\right\}}_{e\in E(n)}$ as context, $g_{\theta }$ predicts $\nabla \tilde{\psi }\left(I\right)$ (51–54). The network consists of context-based normalizations, followed by an attention-based architecture. The detailed architecture is described below.

#### Context aggregation.

With interior equilibrium formulated by Eq. **2**, we represent the local equilibrium state of an interior node n by assembling the set ${\left\{\left(A^{n,e},I^{e}\right)\right\}}_{e\in E(n)}$ into a deformation token, with each pair $\left(A^{n,e},I^{e}\right)$ treated as a deformation subtoken. This physics-informed tokenization yields a modular representation of the underlying stress–strain relationship, allowing tokens from different strain fields, geometries, and loading configurations of the same material to be flexibly assembled into a common physical context.

During training, the physical context for each material is assembled through a hierarchical sampling strategy. First, we construct a candidate pool of strain fields by uniformly sampling the number of involved geometries (1 to 7), loading modes (1 to 3), and loading magnitude within each loading mode (1 to 10) (Fig. 2*A*). Next, 1 to 5 strain fields are randomly drawn from this customized pool. Finally, we uniformly sample 500 to 1,500 deformation tokens from these selected fields to construct the physical context.

During testing, we aggregate all tokens from contextual deformation fields instead of applying random sampling. By retaining the entirety of the available tokens without any information loss, we ensure that ICM fully exploits the most comprehensive physical context possible, thereby maximizing inference accuracy and robustness.

#### Normalization of the coefficient matrices.

Discretized strain fields may involve nonuniform meshes, which can lead to large variations in the magnitude of the coefficient matrices $A^{n,e}$, as $A^{n,e}$ is sensitive to mesh resolution, an ad hoc factor irrelevant to the underlying material response. To eliminate this nonphysical sensitivity, we exploit the linear homogeneity of the interior equilibrium equations (Eq. **2**) to normalize $A^{n,e}$ in a node-wise and direction-wise manner. Specifically, for each node n and spatial direction i, we define the normalized coefficients as

[28] $$\begin{array}{c} \begin{array}{cc} & {\overset{¯}{A}}_{\mathrm{im}}^{n,e}=A_{\mathrm{im}}^{n,e}/\eta_{i}^{n},\;\forall e\in E\left(n\right),\;\forall m\in M,\\ & \eta_{i}^{n}=(\sum_{e^{\prime }\in E\left(n\right)}\sum_{m^{\prime }}{\left(A_{im^{\prime }}^{n,e^{\prime }}\right)}^{2}{)}^{1/2}, \end{array} \end{array}$$

where $\eta_{i}^{n}$ is the local root-sum-square magnitude of the i-th force component at node n. Such normalization suppresses variations caused purely by discretization while preserving the underlying equilibrium presented to the network.

#### Normalization of the strain invariants.

To standardize the range of strain invariants in both contexts and queries, we first center all of them by the strain invariants at the undeformed state $I_{0}={\left(2,1\right)}^{⊤}$, and then normalize them by the root-mean-square deviation from $I_{0}$ calculated over K, i.e., the collection of all node-element pairs (n,e) in the context. This context-based normalization ensures that the input invariants are properly centered and adaptively scaled relative to the contextual deformation state.

#### Attention-based architecture.

Each deformation token is initially mapped to a context embedding in an independent manner. First, for each normalized deformation token, we map its subtokens to latent vectors via a shared linear projection. These vectors are then processed by position-encoding-free self-attention blocks. Finally, we apply average pooling to these vectors to produce a context embedding for each token. Such an embedding is permutation-invariant to subtoken ordering, in line with the physical interpretation that a node’s equilibrium state is independent of the indexing of its neighboring elements.

Next, we facilitate information exchange within the context and enable the query to adaptively aggregate the context. In particular, for each normalized query, we first apply a linear projection to obtain a query embedding. The query and context embeddings are then processed by a stack of attention blocks. In each block, the query embedding attends to the context embeddings to extract the information relevant to the queried invariant state, while the context embeddings are updated by self-attention to capture interactions within the context, with two attention mechanisms sharing the same parameters for simplicity. Again, we apply no positional encoding here, making sure the processed query embeddings are invariant to the ordering of context embeddings. Moreover, since query embeddings do not attend to one another, multiple queries can be evaluated in parallel and independently. Finally, another linear layer is applied to the query embeddings to get the output of $g_{\theta }$.

#### Relation to graph-based learning approaches.

Local aggregation over mesh neighborhoods and permutation invariance are shared with graph-based geometric learning methods (55, 56). In ICM, however, each neighborhood and its associated features are derived directly from the discretized nodal equilibrium equation in Eq. **2**, such that each deformation token represents a local force equilibrium equation. After tokenization, the original mesh adjacency is no longer retained for iterative message passing; instead, tokens from multiple deformation fields, geometries, and loading conditions are assembled as an unordered physical context for constitutive inference.

### Training and Inference.

We train $g_{\theta }$ by enforcing the interior equilibrium constraints in Eq. **2**. At inference, a scaling factor α is applied to the network output to satisfy the boundary conditions in Eq. **5**.

#### Dimensionless loss function.

The predicted nodal force is the assembly of predicted element-wise contributions, i.e.,

[29] $$\begin{array}{c} \begin{array}{cc} {\hat{F}}^{n}\left(\theta \right) & =\sum_{e\in E(n)}{\hat{F}}^{n,e}\left(\theta \right),\\ {\hat{F}}^{n,e}\left(\theta \right) & =A^{n,e}g_{\theta }\left(C\right)\left(I^{e}\right), \end{array} \end{array}$$

where ${\hat{F}}^{n}\left(\theta \right)$ is the predicted nodal force at node n, ${\hat{F}}^{n,e}\left(\theta \right)$ represents the predicted force contribution from element e to node n, and E(n) denotes the set of elements connected to node n.

Directly minimizing the norm of ${\hat{F}}^{n}\left(\theta \right)$ would lead to a trivial collapsing solution, as the network can simply suppress the overall amplitude of the output toward zero to minimize the objective. To circumvent this, we define a dimensionless loss function

[30] $$\begin{array}{c} L\left(\theta \right)=\frac{|N^{\text{in}}{|}^{-1}\sum_{n\in N^{\text{in}}}{∥{\hat{F}}^{n}\left(\theta \right)∥}^{2}}{{\left|K\right|}^{-1}\sum_{(n,e)\in K}{∥{\hat{F}}^{n,e}\left(\theta \right)∥}^{2}}. \end{array}$$

In this formulation, the denominator acts as a self-normalizing factor derived from the context. This objective function is invariant to the scaling of the network output, forcing the network to focus on the physical equilibrium during training.

#### Postscaling.

The interior equilibrium equations in Eq. **2** are homogeneous in the energy gradient ∇ψ(I). As a result, if ∇ψ(I) satisfies the interior equilibrium equations, then so does α∇ψ(I) for any scalar α. In other words, the neural network prediction can only approximate ∇ψ(I) up to a scaling factor. At inference, we determine such a scaling factor from the boundary conditions in Eq. **5**.

In particular, for the j-th boundary condition of the k-th contextual strain field, we obtain the predicted external force

[31] $$\begin{array}{c} {\hat{F}}_{j,k}=\sum_{n\in N_{j,k}^{\text{b}}}{\hat{F}}^{n}, \end{array}$$

where $N_{j,k}^{\text{b}}$ is the corresponding boundary node set. Since the external force is prescribed as a scalar magnitude along the loading direction, the comparison is performed on directional projections rather than Cartesian components. This avoids singular or unstable ratios that may arise when an individual Cartesian component is zero or nearly zero. Accordingly, we calculate the unscaled prediction of external force magnitude as

[32] $$\begin{array}{c} {\hat{f}}_{j,k}={\hat{F}}_{j,k}\cdot d_{j,k}, \end{array}$$

where $d_{j,k}$ is the loading direction. We also use $f_{j,k}$ to denote the corresponding true loading force magnitude calculated in the same way.

By comparing the unscaled prediction and true external forces across all available boundary conditions, a single scaling factor can be determined as

[33] $$\begin{array}{c} \begin{array}{cc} \alpha = & \frac{1}{\left|B\right|}\sum_{(j,k)\in B}\alpha_{j,k},\;\alpha_{j,k}=\frac{{\hat{f}}_{j,k}}{f_{j,k}}. \end{array} \end{array}$$

The final prediction of the energy gradient is then given by

[34] $$\begin{array}{c} \nabla \psi \left(I\right)=\alpha^{-1}\nabla \tilde{\psi }\left(I\right)\approx \alpha^{-1}g_{\theta }\left(C\right)\left(I\right). \end{array}$$

#### Evaluation metrics.

To assess the model performance quantitatively, we introduce the relative prediction error ${\left(\cdot \right)}^{\text{err}}$ between the predicted and true physical quantities. For instance, to evaluate the stress prediction, we calculate

[35] $$\begin{array}{c} S^{\text{err}}=\;\text{average}\left(\sqrt{\sum_{i,j=1}^{2}{\left[\frac{S_{\mathrm{ij}}^{p}-S_{\mathrm{ij}}^{t}}{\max\left(S_{\mathrm{ij}}^{t}\right)-\min\left(S_{\mathrm{ij}}^{t}\right)}\right]}^{2}}\right), \end{array}$$

where the average is taken over the whole mesh. Here, $S_{\mathrm{ij}}^{p}$ and $S_{\mathrm{ij}}^{t}$(i,j=1,2) are the components of the predicted and true stress tensors for a single element, respectively, and the denominator evaluates the range of each component $S_{\mathrm{ij}}^{\text{t}}$ across the mesh.

#### Optimizer and Scheduler.

We optimize the network using the Muon algorithm (57), which performs momentum SGD updates followed by Newton-Schulz orthogonalization on 2D parameter matrices to improve conditioning and generalization. Parameters that are not naturally represented as 2D matrices are optimized with AdamW (58), providing a reliable fallback for remaining components. For the learning rate, we employ a warmup-cosine schedule (59, 60). The learning rate increases linearly from zero to a peak value of $5\times 10^{-4}$ during the first 10% of training steps, and then follows a cosine decay to 10% of the peak value by the final step. This schedule mitigates early instabilities and yields smooth and stable convergence.

## Supplementary Material

Appendix 01 (PDF)

## Data Availability

The code used for model training and analysis is available under an Apache-2.0 license on GitHub at https://github.com/scaling-group/icm-hyperelastic (61). The training and testing datasets are hosted on Hugging Face at https://huggingface.co/datasets/scaling-group/icm-hyperelastic (62).
