# Supplementary material for: In-context learning as a retraining-free paradigm for constitutive modeling
Source: Proc Natl Acad Sci U S A. 2026 Oct 30;123(44):e2620983123. doi: 10.1073/pnas.2620983123 (PMC13732078; doi:10.1073/pnas.2620983123)
Supplement: Supplementary file 1 [file pnas.2620983123.sapp.pdf]

Supporting Information for

In-context learning as a retraining-free paradigm for constitutive modeling

Lingfeng Li, Zhuoyuan Li, Shun Li, Kaixin Zhan, Huajian Gao, Chang Qing Chen, Liu Yang

Corresponding authors: Huajian Gao, Chang Qing Chen, Liu Yang.
E-mail: gao.huajian@tsinghua.edu.cn, chencq@tsinghua.edu.cn, yangliu@nus.edu.sg

This PDF file includes:

Supporting text
Figs. S1 to S4
Tables S1 to S6
SI References

Supporting Information Text

Dataset

Strain energy models. Our datasets cover various isotropic hyperelastic material classes. For isotropic hyperelasticity, the strain energy density function ψ can be expressed as a scalar function of the three strain invariants of the right Cauchy-Green tensor $\boldsymbol{C}$, i.e., $\psi = \psi(I_1, I_2, I_3)$. In this work, we focus on plane strain. The detailed forms of ψ used in this work are listed below.
Polynomial model. The polynomial model is one of the most widely used phenomenological hyperelastic formulations. It defines the strain energy density function as a polynomial expansion of the strain invariants (1, 2). Owing to its flexible form, the model can reproduce a wide range of nonlinear stress-strain relationships through appropriate selection of the polynomial order and material coefficients. In this paper, we use polynomial models up to the sixth order. The strain energy density function is given by

$$\psi = \sum_{k=1}^{6} \sum_{\substack{i,j \geq 0 \\ i+j=k}} C_{ij}(\bar{I}_1 - 3)^i (\bar{I}_2 - 3)^j + \sum_{m=1}^{4} D_m (J-1)^{2m}, \tag{1}$$

where $\bar{I}_1 = J^{-2/3} I_1$ and $\bar{I}_2 = J^{-4/3} I_2$ are isochoric invariants, $J = \det \boldsymbol{F} = \sqrt{\det \boldsymbol{C}}$ is the volume ratio. The material parameters are the polynomial coefficients C_{ij} and D_m for all indices.
Ogden model. The Ogden model represents the strain energy density in terms of principal stretches, using a series of power-law terms (3). It is particularly effective in describing large deformations observed in rubber-like and biological tissues (4, 5). We employ a six-term Ogden model, and the strain energy density function is given by

$$\psi = \sum_{k=1}^{6} \frac{2\mu_k}{\alpha_k^2} (\bar{\lambda}_1^{\alpha_k} + \bar{\lambda}_2^{\alpha_k} + \bar{\lambda}_3^{\alpha_k} - 3) + \sum_{m=1}^{4} D_m (J-1)^{2m}, \tag{2}$$

where $\lambda_1, \lambda_2, \lambda_3$ are the principal stretches, and we set the isochoric principal stretch $\bar{\lambda}_i = J^{-1/3} \lambda_i$ for each $i \in \{1, 2, 3\}$. The material parameters are μ_k, α_k, and D_m for all indices.
Pucci–Saccomandi model. The Pucci–Saccomandi (PS) model refines the Gent model (6) by capturing the limiting-chain stretch of soft materials. It introduces an exponential-type formulation of the invariants, which leads to a logarithmic term in strain energy, and improves stability at large strains (7). In this work, we adopt its compressible form

$$\psi = -\frac{\mu J_{\mathrm{m}}}{2} \ln \left(1 - \frac{\bar{I}_1 - 3}{J_{\mathrm{m}}}\right) + C_2 \ln \left(\frac{\bar{I}_2}{3}\right) + D \left(\frac{J^2 - 1}{2} - \ln J\right), \tag{3}$$

where μ is the shear modulus and J_{m} is the limiting chain stretch. The material parameters are μ, J_{m}, C_2 and D.
Exp-Ln model. The exponential-logarithmic (Exp-Ln) model combines exponential and logarithmic terms in the strain energy function. It features a simple mathematical form, physically meaningful parameters that can be determined from a single uniaxial test, and good agreement with experimental data (8). In this work, we employ its compressible form as

$$\psi = \frac{\mu}{2} \left[\frac{1}{a} \exp[a(\bar{I}_1 - 3)] + b(\bar{I}_1 - 2)[1 - \ln(\bar{I}_1 - 2)] - c\right] + D \left(\frac{J^2 - 1}{2} - \ln J\right). \tag{4}$$

The material parameters are μ, a, b, and D with $c = a^{-1} + b$.
Van der Waals model. The van der Waals model incorporates concepts from statistical mechanics and molecular network theory (9, 10). It captures the finite extensibility of polymer chains by introducing a singularity at the limiting stretch, thereby providing a physically motivated link between macroscopic mechanical behavior and molecular chain extensibility (10). The strain energy density function is expressed as

$$\psi = \mu \left[-(\lambda_{\mathrm{m}}^2 - 3)[\ln(1 - \eta) + \eta] - \frac{2a}{3} \left(\frac{\tilde{I} - 3}{2}\right)^{3/2}\right] + D \left(\frac{J^2 - 1}{2} - \ln J\right), \tag{5}$$

where $\tilde{I} = (1 - \beta)\bar{I}_1 + \beta \bar{I}_2$ and $\eta = \sqrt{(\tilde{I} - 3)/(\lambda_{\mathrm{m}}^2 - 3)}$. The material parameters are μ, λ_{m}, a, β, and D.

Data generation. *Training set.* The training set is based on the polynomial model (eq. (1)) with 31 material parameters. In total, we generated 2,000 materials with various material parameters to construct the training set. More specifically, the training set can be divided into three subsets as follows.

(A) For the first 500 materials, two randomly-selected coefficients of the lower-order ones C_{ij} for $i + j = k \leq 3$ were sampled from a uniform distribution $\mathcal{U}(0, 100)$. The other C_{ij} coefficients are set to zero. Two of the volumetric coefficients (D_1, D_2, D_3, D_4) are randomly selected and sampled from $\mathcal{U}(0, 100)$, while the remaining two were set to zero. A post-sampling shift was applied by increasing both C_{10} and D_1 by 1;

(B) For the next 500 materials, four randomly-selected C_{ij} coefficients were sampled from a uniform distribution $\mathcal{U}(0, 100)$. The other C_{ij} coefficients are set to zero. The same sampling strategy for the D_m coefficients is applied as above.

(C) For the remaining 1,000 materials, the coefficients C_{ij} and D_m are sampled from $\mathcal{U}(0, 100)$ for all indices.

To ensure numerical stability and comparable stress magnitudes among different models, the generated polynomial coefficients undergo a two-step normalization process as follows.

- Basis-function standard deviation normalization: each basis function may contribute to the stress with a different magnitude. To balance their relative influence, we first compute the standard deviation of the stress response for each individual basis function under 1000 random 2×2 deformation gradients. The coefficients are then divided by these standard deviations, ensuring that all basis functions produce comparable stress amplitudes.
- Tangent stiffness normalization: even after the first step, different models can have very different initial stiffnesses. To standardize the small-strain response, we evaluate the stress difference under a small uniaxial compression and tension ($F_{11} = 0.9$ and $F_{11} = 1.1$ with fixed $F_{22} = 1.0$) and estimate the effective tangent stiffness $\partial P_{11}/\partial F_{11}$. Each model's coefficients are then divided by the stiffness so that all models have similar initial stiffness and stress levels.

Test set. A total of four test sets, denoted as Test-ID (in distribution), Test-M (new materials), Test-MGL (new materials, geometries and loadings) and Test-MGL+ (new materials, geometries and extended loadings) are generated to comprehensively validate the model performance.

We generated the Test-ID set consisting of 400 materials with the polynomial model eq. (1). The test set comprises three subsets of size $(100, 100, 200)$ and then is sampled with the same strategy as the training set.

The Test-M set evaluates out-of-distribution generalization by excluding polynomial models entirely. Instead, it consists of alternative hyperelastic formulations: the Ogden, Pucci–Saccomandi, Exp-Ln, and van der Waals models. Specifically, for the Ogden model defined in eq. (2), we generated 200 parameter configurations divided into two complexity levels as below.

(A) The first 100 materials are low-order models, where we set $\mu_k = 0$ for $k \geq 3$ and the remaining shear moduli μ_k were sampled from $\mathcal{U}(1, 101)$. To determine the exponent α_k, we sampled $x \sim \mathcal{N}(0, 1)$ and set α_k as the clipping of $(2|x| + 1)$ within the interval $[1.2, 20]$. All volumetric coefficients D_m were sampled from $\mathcal{U}(0, 100)$. A post-sampling shift was applied by increasing D_1 by 1 to ensure a nonzero small-strain volumetric stiffness;

(B) The remaining 100 materials are high-order models, where all the shear moduli μ_k were sampled from $\mathcal{U}(1, 101)$, $1 \leq k \leq 6$. The exponents α_k were drawn similarly, but with a scaling factor of 10 instead of 2. The four volumetric coefficients D_m were sampled with the same procedure, and the post-sampling shift also applies.

To complete the Test-M set, 100 materials were generated with the material parameters randomly sampled from the ranges listed in tables S1 to S3 for the Pucci–Saccomandi, Exp-Ln, and van der Waals models, respectively.

Quantifying constitutive response distribution shift. Different analytical forms do not necessarily imply out-of-distribution constitutive responses, because different material models may produce similar stress responses over a bounded deformation range. A quantitative characterization of the distance between the training and test constitutive responses is therefore required. Since the ICM training set is generated using a sixth-order polynomial strain energy function, we examine whether the responses of the five material families in the test sets can be represented within the corresponding polynomial constitutive space.

We sample the deformation and stress responses from the Polynomial materials in Test-ID and the Exp-Ln, Pucci-Saccomandi, Ogden, and van der Waals materials in Test-MGL+. For each geometry-loading case, 100 elements are randomly selected. For the q-th sampled state, $\boldsymbol{F}_q$ denotes the deformation gradient and the corresponding first Piola-Kirchhoff stress is $\boldsymbol{P}_q^{\mathrm{fem}}$. To determine whether these responses can be represented by the polynomial model used for training, we consider polynomial spaces

$$\psi_d = \sum_{k=1}^{d} \sum_{\substack{i,j\geq 0\\ i+j=k}} C_{ij}(\bar{I}_1 - 3)^i(\bar{I}_2 - 3)^j + \sum_{m=1}^{4} D_m(J-1)^{2m} \quad [6]$$

of orders $d = 2, 4,$ and 6. The four volumetric basis functions are retained at all three polynomial orders. Consistent with the generation of the training materials, all coefficients are constrained to be nonnegative. For a given deformation state $\boldsymbol{F}_q$, the stress predicted by the polynomial model is calculated as $\boldsymbol{P}_d(\boldsymbol{F}_q; \boldsymbol{c}_d) = \partial\psi_d/\partial\boldsymbol{F}_q$, where $\boldsymbol{c}_d$ collects all polynomial coefficients. For each material, the optimal coefficients are obtained by solving

$$\boldsymbol{c}_d^* = \arg\min_{\boldsymbol{c}_d\geq 0} \sum_{q=1}^{N} \left\| \boldsymbol{P}_d(\boldsymbol{F}_q; \boldsymbol{c}_d) - \boldsymbol{P}_q^{\mathrm{fem}} \right\|_{\mathrm{F}}^2, \quad [7]$$

where N is the total number of sampled deformation states and $\|\cdot\|_{\mathrm{F}}$ denotes the Frobenius norm. The projected stress is then defined as $\boldsymbol{P}_q^{\mathrm{proj}} = \boldsymbol{P}_d(\boldsymbol{F}_q; \boldsymbol{c}_d^*)$.

The relative projection error P^{err} is calculated as

$$P^{\mathrm{err}} = \left(\frac{\sum_{q=1}^{N} \left\| \boldsymbol{P}_q^{\mathrm{fem}} - \boldsymbol{P}_q^{\mathrm{proj}} \right\|_{\mathrm{F}}^2}{\sum_{q=1}^{N} \|\boldsymbol{P}_q^{\mathrm{fem}}\|_{\mathrm{F}}^2} \right)^{1/2}. \quad [8]$$

Figure S1 shows the resulting projection errors. At second order, the median errors of all five material families are relatively large, indicating that the reduced polynomial space is insufficient to represent most of their responses. As the polynomial order increases, the median error of the Polynomial Test-ID materials decreases substantially and reaches the order of 10^{-5} at sixth order, confirming that responses generated by the training polynomial family can be accurately recovered by the projection.

By contrast, at sixth order, the median projection errors of all four non-polynomial material families are more than three orders of magnitude larger than the corresponding error for the Polynomial materials. This pronounced separation demonstrates that, over the sampled deformation domain, their constitutive responses cannot be accurately represented by the sixth-order polynomial space used to generate the training set.

The sixth-order projection space considered here contains all nonnegative combinations of the polynomial basis functions and is therefore broader than the finite collection of 2,000 polynomial materials included in the training set. The projection errors of the non-polynomial materials therefore provide the characterization of their separation from the training response space. These results clarify that out-of-distribution material generalization in this work refers not merely to the use of different analytical material models, but to constitutive responses that cannot be accurately represented within the training polynomial space over the deformation domain examined.

Comparison with the ENN model

The Equilibrium-based Neural Network (ENN) (11, 12) provides a physics-informed baseline for learning a hyperelastic stress-strain relationship from full-field deformation measurements. Similar to the proposed ICM, the ENN leverages strain fields together with force equilibrium constraints and does not require stress labels. A key difference is that the ENN is trained for a single material using measured external forces, and thus does not transfer to unseen materials without retraining, whereas the ICM is designed for cross-material stress inference from contextual strain fields.

ENN parameterization and invariance. In the plane strain condition, the strain energy density function ψ is parameterized as a scalar function of the invariants (I_1, I_3). The ENN takes these two invariants as inputs and predicts $\psi_{\boldsymbol{\theta}}$ at each material point. The partial derivatives $\partial\psi/\partial I_m$ $(m = 1, 3)$ are evaluated by automatic differentiation and are used to assemble the corresponding stresses and interior nodal forces. Material isotropy is embedded by expressing $\psi_{\boldsymbol{\theta}}$ in terms of invariants.

Architecture modification. The original ENN implementation uses a convolutional neural network (CNN) consisting of a stack of 1×1 convolutional layers to map strain component inputs to the stress component outputs. Since a 1×1 convolution performs an independent channel-wise affine transformation at each spatial location, it is equivalent to applying the same multilayer perceptron (MLP) to every material point. In our implementation, we therefore replace the 1×1 convolutional layers with an explicit MLP that takes the invariant pair (I_1, I_3) as input and outputs the strain energy density $\psi_{\boldsymbol{\theta}}(I_1, I_3)$.

This change is purely notational and implementation-oriented, and does not change the functional form of the ENN model. It streamlines the evaluation of $\psi_{\boldsymbol{\theta}}(I_1, I_3)$ and its automatic-differentiation derivatives, and reduces architectural differences between ENN and the proposed ICM. The comparison thus primarily reflects differences in stress inference accuracy and deployment time.

Equilibrium constraints and loss function. The strain fields are discretized into finite elements to compute nodal forces $\boldsymbol{f}_n(\boldsymbol{\theta})$. For interior nodes, force equilibrium requires $\boldsymbol{f}_n = \mathbf{0}$. For boundary nodes, we enforce global equilibrium by matching the predicted boundary resultant forces to the experimentally measured external forces. Specifically, for each displacement-controlled boundary indexed by i, the predicted resultant force is computed by summing the nodal forces on that boundary as

$$\hat{\boldsymbol{F}}_{b_i}(\boldsymbol{\theta}) = \sum_{n \in \mathcal{N}_{b_i}} \boldsymbol{f}_n(\boldsymbol{\theta}), \tag{9}$$

and the prediction is compared with the measured resultant force $\boldsymbol{F}_{b_i}$.

The training loss of the ENN is

$$L = L_{\text{in}} + w_{\text{b}} L_{\text{b}} \tag{10}$$

as a weighted sum of the interior equilibrium loss L_{in} and the boundary loss L_{b} with a weight w_{b}. The interior equilibrium loss L_{in} is defined as

$$L_{\text{in}} = \frac{1}{N_{\text{free}}} \sum_{n \in \mathcal{N}_{\text{free}}} l(\boldsymbol{f}_n(\boldsymbol{\theta}), \mathbf{0}), \tag{11}$$

where N_{free} is the total number of interior nodes and $l(\cdot, \cdot)$ is the vector Huber loss evaluated by summing up the loss for each component as

$$l(\boldsymbol{x}, \boldsymbol{y}) = \sum_k H(x_k, y_k), \qquad H(x, y) = \begin{cases} \frac{1}{2}(x - y)^2, & \text{if } |x - y| < 1, \\ |x - y| - \frac{1}{2}, & \text{if } |x - y| \geq 1. \end{cases} \tag{12}$$

The boundary loss L_{b} is defined by averaging over all displacement boundaries as

$$L_{\text{b}} = \frac{1}{N_{\text{b}}} \sum_{i=1}^{N_{\text{b}}} L_{b_i}, \quad L_{b_i} = l\left(\frac{\hat{\boldsymbol{F}}_{b_i}(\boldsymbol{\theta})}{N_{b_i}}, \frac{\boldsymbol{F}_{b_i}}{N_{b_i}} \right), \tag{13}$$

where N_b is the number of displacement boundaries and N_{b_i} is the number of nodes on boundary i. The normalization by N_{b_i} prevents the boundary term from being sensitive to the boundary node count.

Normalization and force scaling. The inputs (I_1, I_3) are normalized using the mean and standard deviation computed from all training data points. To improve numerical stability, we also scale the nodal forces by a characteristic force magnitude estimated from the training set. Specifically, we define a force scale s_f as the average of the absolute values of the measured external forces across all training samples, and divide all predicted nodal forces and boundary resultants by s_f when evaluating eqs. (11) and (13), which yields a dimensionless training objective and reduces sensitivity to the overall force magnitude.

Model configurations and training details. We trained the ENN models with a set of strain fields and measured boundary external forces by minimizing L in eq. (10), and three different model configurations summarized in table S4 were considered. The boundary loss weighting coefficient w_b was fixed at 10^{-1}. We trained the neural networks using the Adam optimizer with an initial learning rate of 10^{-3}. We applied a StepLR learning-rate schedule, reducing the learning rate by a factor of 0.95 every 100 optimization steps.

Effect of the number of observed deformation fields. We further investigated how the number of deformation fields observed for each target material affects the accuracy and deployment cost of the ENN and ICM. To ensure a controlled comparison, both methods used models containing approximately 4 million parameters. For each of the 500 materials, which are common to Test-M and Test-MGL+, we selected the same K deformation fields from Test-M, with $K = 1$, 5, or 10. These fields were used as the material-specific training set for the ENN and as the context fields for ICM. The two methods were subsequently evaluated on the same fields from Test-MGL+. Therefore, the ENN and ICM were provided with the same material observations, while differing only in how this information was incorporated. The ENN learned a separate material-specific constitutive model through optimization, whereas ICM directly conditioned its prediction on the context fields without parameter optimization.

As shown in fig. S2, increasing the number of context fields consistently improves the accuracy of ICM. Its geometric mean relative stress error decreases from 6.27×10^{-2} for $K = 1$ to 2.30×10^{-2} for $K = 5$ and 1.82×10^{-2} for $K = 10$. This improvement is accompanied by an increase in the mean inference time from approximately 15.0 ms to 56.1 ms and 122.2 ms, respectively.

For the ENN, increasing K has two competing effects under a fixed deployment time budget. Additional deformation fields provide richer mechanical information, but they also increase the computational cost of each optimization epoch. At a deployment time of approximately 100 s, the geometric mean relative stress errors are 5.45×10^{-2}, 3.91×10^{-2}, and 5.12×10^{-2} for $K = 1$, 5, and 10, respectively. Thus, $K = 5$ provides the best ENN accuracy within the considered time range, whereas increasing the training set to $K = 10$ does not lead to a further improvement within the same computational budget. In contrast, ICM can exploit additional observations directly during inference and therefore shows a monotonic improvement with increasing K.

Experimental setup

In addition to the plane strain setting used throughout this work, we conducted an auxiliary training experiment under plane stress. The ICM training and the hyperparameter settings are the same as those for the plane strain case, and the only difference was that the stress-strain relationship is constrained by the plane stress condition. This is the only experiment in this study that adopts the plane stress assumption.

Materials. Two hyperelastic materials with different stiffnesses were used in the experiments. For clarity, we refer to them as the soft and hard materials. All specimens were fabricated by Wenext Factory (China) and characterized by large reversible deformations owing to their high elasticity.

Specimen geometry and DIC measurements. We designed three distinct specimen geometries, including configurations with geometric imperfections, to induce non-uniform deformation fields and thereby increase the diversity of the contextual strain fields (fig. S3). For each test, full-field deformation data were obtained and subsequently processed into deformation tokens that serve as the context input for model validation. All specimens were subjected to uniaxial tensile loading, during which full-field deformation data were captured using a digital image correlation system. A random black-and-white speckle pattern was sprayed on the specimen surfaces to ensure optical tracking. Images were recorded at two frames per second, while the specimens were loaded at a displacement rate of 22.5 mm/min up to a total displacement of 40 mm. This setup enabled accurate measurement of displacement and strain fields across the entire specimen surface.

The acquired full-field deformation data allowed the ICM to infer the thickness-averaged stress in the specimen under the following assumptions. We denote the three-dimensional stress–strain relationship by $\boldsymbol{P} = \boldsymbol{P}(\boldsymbol{F})$, where both the first Piola–Kirchhoff stress $\boldsymbol{P}$ and deformation gradient tensor $\boldsymbol{F}$ are three-dimensional second-order tensors. Because the specimens are thin relative to their in-plane dimensions and both free surfaces are traction-free, we adopted a plane stress approximation and assumed that the stress and deformation vary weakly through the thickness. Under the assumption, the out-of-plane shear components of both stress and strain vanish, and the out-of-plane normal stress is zero. Accordingly, $\boldsymbol{F}$ and $\boldsymbol{P}$ take the forms

$$[\boldsymbol{F}] = \begin{bmatrix} F_{xX} & F_{yX} & 0 \\ F_{xY} & F_{yY} & 0 \\ 0 & 0 & \lambda_3 \end{bmatrix}, \quad [\boldsymbol{P}] = \begin{bmatrix} P_{xX} & P_{yX} & 0 \\ P_{xY} & P_{yY} & 0 \\ 0 & 0 & 0 \end{bmatrix}, \tag{14}$$

where λ_3 represents the stretch in the thickness direction. The surface deformation measurements determine the in-plane components of $\boldsymbol{F}$, denoted as $\boldsymbol{F}_{2\mathrm{D}}$, and we define $\boldsymbol{P}_{2\mathrm{D}}$ as

$$[\boldsymbol{F}_{2\mathrm{D}}] = \begin{bmatrix} F_{xX} & F_{yX} \\ F_{xY} & F_{yY} \end{bmatrix}, \quad [\boldsymbol{P}_{2\mathrm{D}}] = \begin{bmatrix} P_{xX} & P_{yX} \\ P_{xY} & P_{yY} \end{bmatrix}. \qquad [15]$$

The proposed ICM model learns the two-dimensional stress–strain relationship $\boldsymbol{P}_{2\mathrm{D}} = \boldsymbol{P}_{2\mathrm{D}}(\boldsymbol{F}_{2\mathrm{D}})$, which can be interpreted as the three-dimensional stress–strain relationship $\boldsymbol{P} = \boldsymbol{P}(\boldsymbol{F})$ constrained by the plane stress condition $P_{zZ}(\boldsymbol{F}_{2\mathrm{D}}, \lambda_3) = 0$. Under the assumption of uniform through-thickness deformation, we can average the three-dimensional equation of stress equilibrium over the thickness direction to yield the in-plane equilibrium

$$\frac{\partial P_{xX}}{\partial X} + \frac{\partial P_{xY}}{\partial Y} = 0 \qquad [16]$$

$$\frac{\partial P_{yX}}{\partial X} + \frac{\partial P_{yY}}{\partial Y} = 0 \qquad [17]$$

Based on $\boldsymbol{F}_{2\mathrm{D}}$ and $\boldsymbol{P}_{2\mathrm{D}}$, nodal forces can be computed in the same way as in the simulation setting, and the nodal force equilibrium condition used for training remains applicable. Moreover, we used two invariants derived from the in-plane deformation, denoted as $I_1^{2\mathrm{D}}$ and $I_3^{2\mathrm{D}}$, as the ICM input. Here, $I_1^{2\mathrm{D}}$ and $I_3^{2\mathrm{D}}$ are computed from $\boldsymbol{F}_{2\mathrm{D}}$. Under plane stress, only two invariants are independent, and the remaining invariant can be derived from $I_1^{2\mathrm{D}}$ and $I_3^{2\mathrm{D}}$.

The total forces recorded by the uniaxial tension machine were divided by the initial thicknesses and assigned to the boundary total forces during the ICM inference. Although the normal strain and stress through the thickness were not directly measured, they are not required for training or inferring the in-plane stress under the plane stress assumption.

Preprocessing of experimental data. DIC provides the surface coordinates and displacements of tracked points on a regular, pixel-style grid. We denoised and interpolated the measured displacement field onto a set of triangular meshes using a radial basis function (RBF) interpolator (13) with a thin-plate-spline kernel and a linear polynomial tail (degree 1). The target meshes resembled the meshes used during training of the ICM model. We computed a characteristic point spacing h from the DIC points as the median distance to each point's 7th nearest neighbor (computed in the 2D coordinate space). The smoothing parameter of the interpolator was chosen as $0.05h^2$. This resulted in good interpolation in well-sampled interior regions, suppressed measurement noise and stable extrapolation where the domain was not fully covered by DIC data (e.g., the boundaries). For computational efficiency, the interpolation at each mesh node used a fixed-size local neighborhood (here 100 nearest DIC points).

To ensure reliable inference, we identified mesh nodes where the interpolated displacements are likely to be inaccurate due to insufficient local DIC support. Specifically, we computed a reference neighborhood radius h_n as the median distance to the $n_e = 8$ nearest neighbor among the DIC points. A mesh node was marked reliable if at least $n_e/2 = 4$ DIC points were located within a radius h_n of that node; otherwise it was marked unreliable. During ICM inference, deformation tokens were constructed only for the reliable nodes and used as the context.

Consistency of experimental validation. To assess the sensitivity of ICM inference to the choice of context geometry, we performed an additional validation study in which the measured full-field deformation data from each specimen geometry were separately converted into deformation tokens and used as the context input to predict the uniaxial tensile response (fig. S4). The predicted nominal stress responses obtained from these three contexts are highly consistent, indicating that the model provides stable stress predictions despite specimen-to-specimen variations and differences in the geometry used to construct the context.

Quantitative analysis of context embeddings

In the main text, we visualized the high-dimensional context embeddings produced by ICM for deformation fields drawn from the four test sets using a two-dimensional t-SNE projection. Each point in this visualization represents a context embedding constructed from a particular deformation field. Consequently, the embedding may encode constitutive response information together with information associated with the deformation coverage, geometry, and loading condition of the input field.

The t-SNE projection provides a qualitative visualization suggesting that some nearby embeddings exhibit similar scale-normalized strain energy profiles. However, because t-SNE can distort distances and neighborhood structure during dimensionality reduction, we do not use distances in the two-dimensional projection as quantitative evidence of constitutive similarity. The material models, geometries, loading conditions, and parameters of the representative cases highlighted in Fig. 5 of the main text are summarized in table S5. A quantitative test in the original embedding space is presented below.

Sampling of embeddings. The candidate pool consisted of all original 192-dimensional context embeddings underlying the t-SNE visualization in the main text. From this pool, we sampled 400 query embeddings, with 80 samples from each of the five material classes: Polynomial, Exp-Ln, Pucci-Saccomandi, Ogden, and van der Waals. The Polynomial samples were drawn from Test-ID. For each of the other four material classes, 40 samples were drawn from Test-M and 40 from the combined Test-MGL/Test-MGL+ geometry and loading configurations. Each query embedding was associated with a distinct material.

For each query embedding, Euclidean distances to the candidate embeddings were calculated directly in the original 192-dimensional space. We retained the five nearest embeddings while requiring the five neighbors to represent distinct materials. Two exclusion strategies were considered: (i) same-material exclusion, in which all candidate embeddings associated with the same material as the query were excluded, while embeddings from other materials within the same material class remained eligible; and (ii) same-class exclusion, in which all candidate embeddings belonging to the same material class as the query were excluded. The second strategy provides a more stringent test of whether local proximity is associated with constitutive similarity across different analytical forms of the strain energy function.

To establish a random reference, we randomly selected 100 distinct materials represented in the same candidate pool for each query embedding, using the corresponding exclusion strategy. For each randomly selected material, one of its available embeddings was selected at random, and the associated deformation field was used as the comparison field. This reference was used to assess whether materials associated with nearby embeddings exhibit greater constitutive similarity than expected from random selection.

Scale-normalized constitutive response distance. We quantified constitutive similarity using the strain energy gradient as the primary response signature and the strain energy profile as a complementary signature. Here, m denotes a material model, r denotes a deformation field, $\boldsymbol{I}_{r,i} = (I_{1,r,i}, I_{3,r,i})$ denotes the i-th deformation state in field r, and N_r is the total number of deformation states in that field. The strain energy gradient was defined as

$$\boldsymbol{g}_m(\boldsymbol{I}) = \left[\frac{\partial \psi_m}{\partial I_1}, \frac{\partial \psi_m}{\partial I_3}\right]. \qquad [18]$$

For material m evaluated over field r, the energy values were normalized by their root-mean-square magnitude:

$$\widehat{\psi}_{m,r}(\boldsymbol{I}_{r,i}) = \frac{\psi_m(\boldsymbol{I}_{r,i})}{\left[N_r^{-1}\sum_{j=1}^{N_r}\psi_m(\boldsymbol{I}_{r,j})^2\right]^{1/2}}. \qquad [19]$$

The two components of the energy gradient were normalized together in the same manner:

$$\widehat{\boldsymbol{g}}_{m,r}(\boldsymbol{I}_{r,i}) = \frac{\boldsymbol{g}_m(\boldsymbol{I}_{r,i})}{\left[(2N_r)^{-1}\sum_{j=1}^{N_r}\|\boldsymbol{g}_m(\boldsymbol{I}_{r,j})\|_2^2\right]^{1/2}}. \qquad [20]$$

These normalizations remove the overall response magnitude, allowing the comparison to focus on constitutive response profiles within the deformation range represented by each field.

For two materials m_a and m_b, their normalized energy and gradient distances over field r were defined as

$$\delta_r^{\psi}(m_a, m_b) = \left[\frac{1}{N_r}\sum_{i=1}^{N_r}\left(\widehat{\psi}_{m_a,r}(\boldsymbol{I}_{r,i}) - \widehat{\psi}_{m_b,r}(\boldsymbol{I}_{r,i})\right)^2\right]^{1/2}, \qquad [21]$$

and

$$\delta_r^{g}(m_a, m_b) = \left[\frac{1}{2N_r}\sum_{i=1}^{N_r}\|\widehat{\boldsymbol{g}}_{m_a,r}(\boldsymbol{I}_{r,i}) - \widehat{\boldsymbol{g}}_{m_b,r}(\boldsymbol{I}_{r,i})\|_2^2\right]^{1/2}. \qquad [22]$$

Let m_s denote the material associated with query embedding s, and let r_s and r_c denote the deformation fields associated with the query and selected comparison embeddings, respectively. The final symmetric distances were calculated as

$$d_s^{\psi}(m) = \frac{1}{2}\left[\delta_{r_s}^{\psi}(m_s, m) + \delta_{r_c}^{\psi}(m_s, m)\right], \qquad d_s^{g}(m) = \frac{1}{2}\left[\delta_{r_s}^{g}(m_s, m) + \delta_{r_c}^{g}(m_s, m)\right]. \qquad [23]$$

Neighbor-to-random ratios. For each query embedding and each exclusion strategy, we calculated four quantities: the mean symmetric energy distance to the five nearest neighbors, the mean symmetric energy distance to the 100 random materials, and the corresponding two mean gradient distances. The energy and gradient ratios were then defined separately as

$$R_s^{\psi} = \frac{\overline{d}_{s,\text{neighbor}}^{\psi}}{\overline{d}_{s,\text{random}}^{\psi}}, \qquad R_s^{g} = \frac{\overline{d}_{s,\text{neighbor}}^{g}}{\overline{d}_{s,\text{random}}^{g}}. \qquad [24]$$

The quantities $\overline{d}_{s,\text{neighbor}}^{\psi}$ and $\overline{d}_{s,\text{random}}^{\psi}$ are the mean symmetric energy distances to the five nearest neighbors and the 100 random materials, respectively, while $\overline{d}_{s,\text{neighbor}}^{g}$ and $\overline{d}_{s,\text{random}}^{g}$ are the corresponding mean symmetric gradient distances.

Values of $R_s^{\psi} < 1$ or $R_s^{g} < 1$ indicate that the nearest neighbors have more similar energy profiles or energy gradient responses than randomly selected materials under the same exclusion strategy. The two ratios were calculated separately for each of the 400 query embeddings and then summarized with equal weight across the sampled embeddings.

Results. As summarized in table S6, under same-material exclusion, the mean gradient and energy ratios were 0.150 and 0.102, respectively. When the entire material class was excluded, the corresponding ratios were 0.363 and 0.327. All four mean ratios remained substantially below one, indicating that materials associated with nearby embeddings tend to exhibit more similar scale-normalized constitutive responses than randomly selected materials when both associated deformation fields are considered.

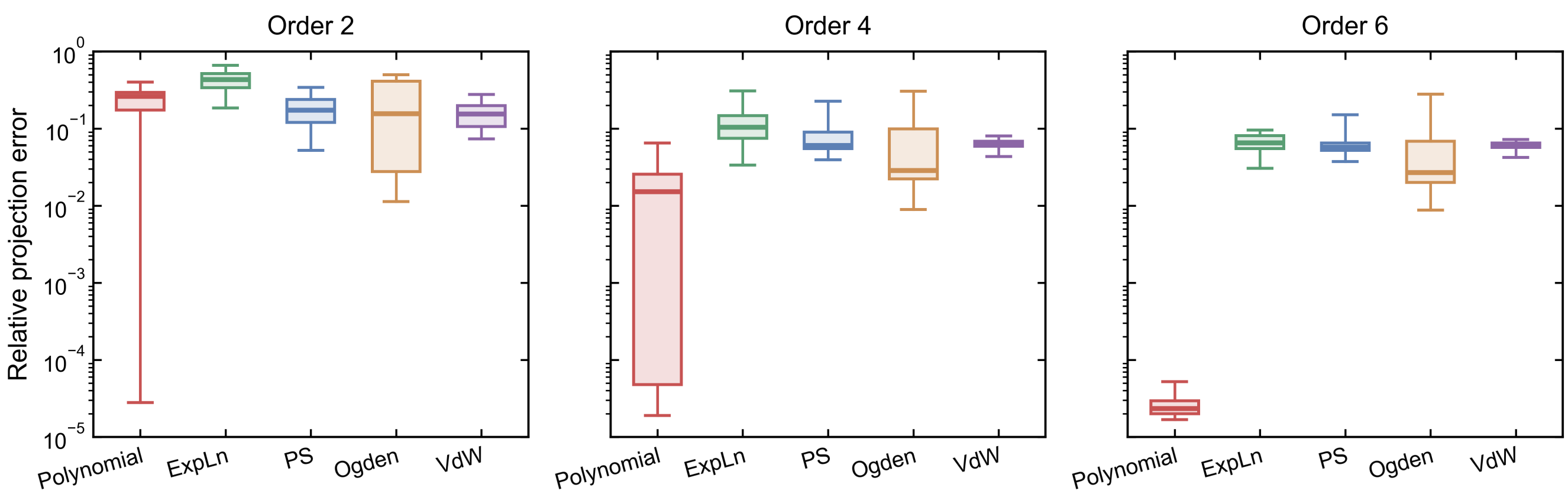

Fig. S1. Relative errors obtained by projecting the constitutive responses of the Polynomial materials in Test-ID and the four non-polynomial material families in Test-MGL+ onto nonnegative polynomial spaces of orders 2, 4, and 6. At sixth order, the Polynomial responses are recovered to the numerical consistency level, whereas the projection errors of all four non-polynomial families remain more than three orders of magnitude larger.

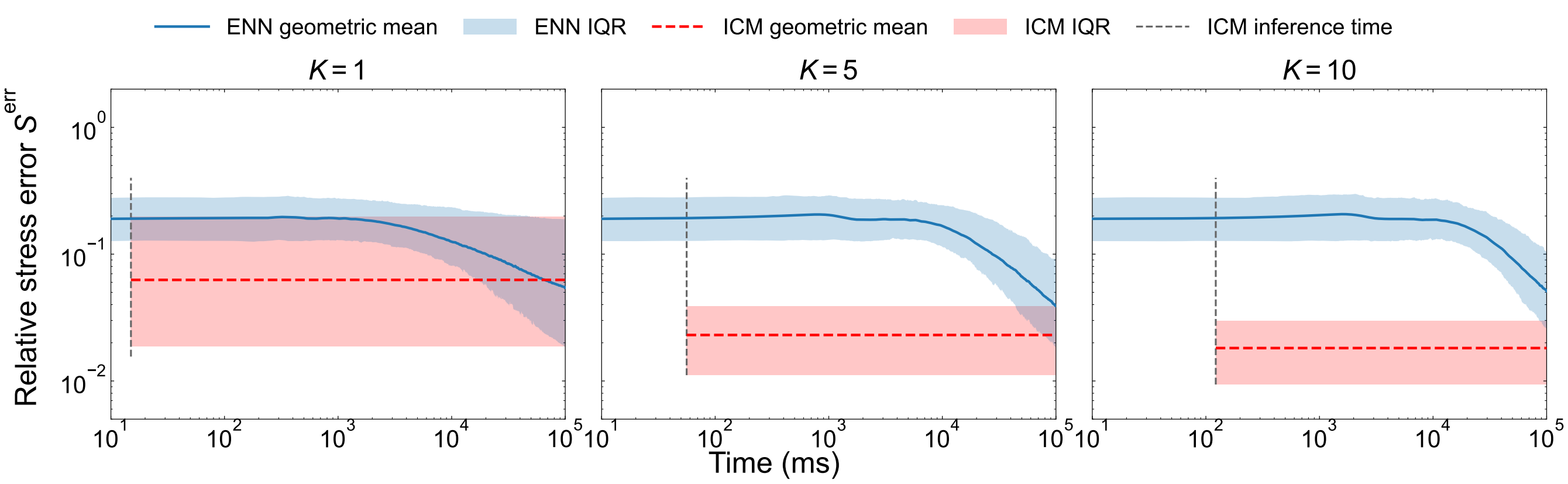

Fig. S2. Effect of the number of observed deformation fields on the ENN and ICM. Both methods contain approximately 4 million parameters. For each material, the same K fields from Test-M are used as the material-specific training set for the ENN and as context for the ICM, while the predictions are evaluated on deformation fields from Test-MGL+. The three panels correspond to $K = 1$, 5, and 10. Curves and horizontal lines denote geometric mean relative stress errors, and the shaded regions indicate the corresponding interquartile ranges. The vertical dashed lines indicate the mean ICM inference time.

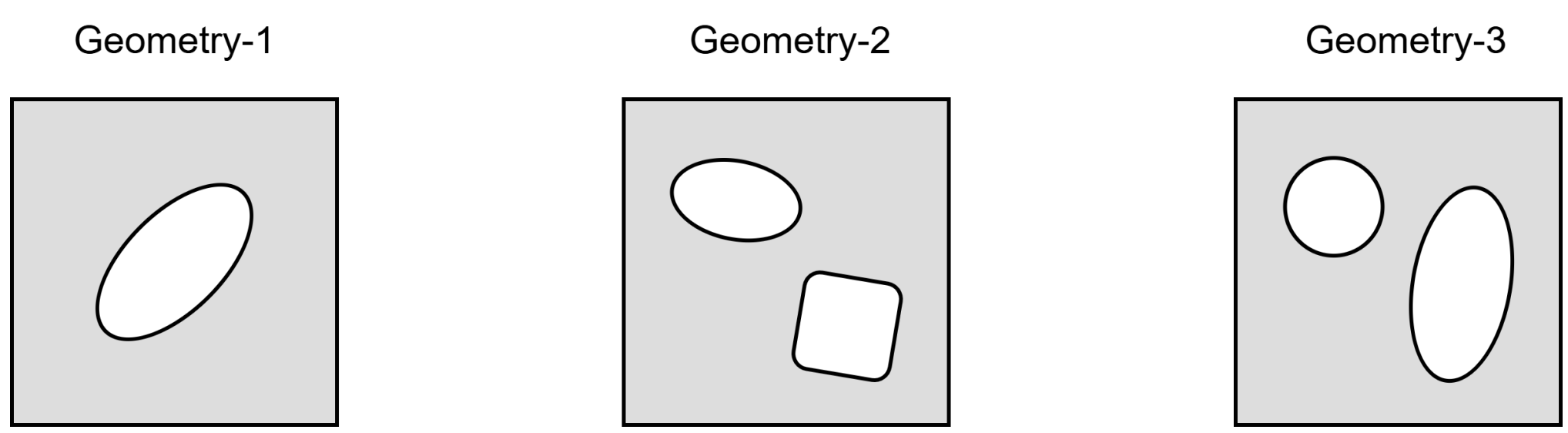

Fig. S3. Three specimen geometries used in the experiments.

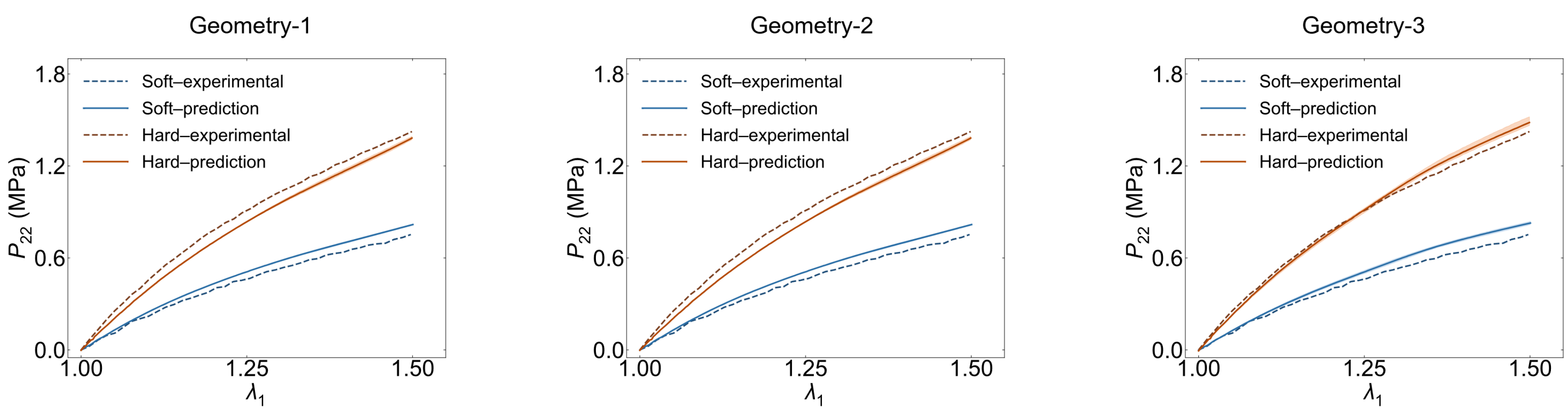

Fig. S4. Predicted uniaxial nominal stress responses using the measured full-field deformation data of three geometries as context.

Table S1. Sampling distributions of the Pucci–Saccomandi model.

Parameter	Description	Distribution
μ (MPa)	Shear modulus	$\mu \sim \mathcal{U}(1, 101)$
J_{m}	Chain stretch limit	$\sqrt{J_{\mathrm{m}}} \sim \mathcal{U}(4, 6)$
C_2	Second invariant coefficient	$C_2 \sim \mathcal{U}(0, 100)$
D (MPa)	Volumetric penalty coefficient	$D \sim \mathcal{U}(1, 501)$

Table S2. Sampling distributions of the Exp-Ln hyperelastic model.

Parameter	Description	Distribution
μ (MPa)	Shear modulus	$\mu \sim \mathcal{U}(1, 101)$
a	Exponential coefficient	$a \sim \mathcal{U}(0.1, 3.1)$
b	Logarithmic coefficient	$b \sim \mathcal{U}(0, 1)$
D (MPa)	Volumetric penalty coefficient	$D \sim \mathcal{U}(1, 501)$

Table S3. Sampling distributions of the van der Waals model.

Parameter	Description	Distribution
μ (MPa)	Shear modulus	$\mu \sim \mathcal{U}(1, 101)$
λ_{m}	Chain stretch limit	$\lambda_{\mathrm{m}} \sim \mathcal{U}(4, 6)$
a	Nonlinearity coefficient	$a \sim \mathcal{U}(0, 0.5)$
β	Weighting factor between I_1 and I_2	$\beta \sim \mathcal{U}(0, 1)$
D (MPa)	Volumetric penalty coefficient	$D \sim \mathcal{U}(1, 501)$

Table S4. The ENN model configurations used for comparison

Model	Layers	Hidden size	Params
Small	2	256	132K
Medium	8	768	4.14M
Large	8	1024	8.40M

Table S5. Representative material models corresponding to the highlighted t-SNE points.

No.	Mat.	Geo.	Loading	Parameters
1	VdW	II	shear<br>$\bar{u}_1 = 0,\ \bar{u}_2/L = 0.15$	$\mu = 32.3797, \quad \lambda_\mathrm{m} = 4.3952,$<br>$a = 0.0596, \quad \beta = 0.4010,$<br>$D = 45.0786$
2	PS	II	shear<br>$\bar{u}_1 = 0,\ \bar{u}_2/L = 0.15$	$\mu = 45.5183, \quad J_\mathrm{m} = 32.1880,$<br>$C_2 = 91.1765, \quad D = 104.2352$
3	Exp-Ln	VI	biaxial<br>$\bar{u}_1/L = 0.5,\ \bar{u}_2/L = 0.125$	$\mu = 5.4041 \times 10^1, \quad a = 3.0775 \times 10^{-1},$<br>$b = 9.3254 \times 10^{-1}, \quad D = 4.4402 \times 10^2$
4	VdW	IV	biaxial<br>$\bar{u}_1/L = 0.5,\ \bar{u}_2/L = 0.5$	$\mu = 18.6790, \quad \lambda_\mathrm{m} = 4.2527,$<br>$a = 4.7719 \times 10^{-1}, \quad \beta = 2.0768 \times 10^{-1},$<br>$D = 3.6567 \times 10^2$
5	PS	IV	biaxial<br>$\bar{u}_1/L = 0.5,\ \bar{u}_2/L = 0.3125$	$\mu = 6.5509, \quad J_\mathrm{m} = 17.1022,$<br>$C_2 = 6.3923, \quad D = 339.7878$
6	Ogden	X	proportional biaxial (2-side)<br>$\bar{u}_1/L = 0.18,\ \bar{u}_2/L = 0.09$	$\mu_1 = 11.6752, \quad \mu_2 = 15.7796,$<br>$\alpha_1 = 4.6862, \quad \alpha_2 = 2.6835,$<br>$D_1 = 54.3825, \quad D_2 = 58.4697,$<br>$D_3 = 75.5499, \quad D_4 = 29.5771$
7	VdW	X	equal biaxial<br>$\bar{u}_1/L = 0.14,\ \bar{u}_2/L = 0.14$	$\mu = 66.5179, \quad \lambda_\mathrm{m} = 4.6732,$<br>$a = 2.1922 \times 10^{-1}, \quad \beta = 4.5983 \times 10^{-1},$<br>$D = 1.1403 \times 10^2$
8	Exp–Ln	IV	uniaxial<br>$\bar{u}_1/L = 0.3,\ \bar{u}_2 = 0$	$\mu = 40.7137, \quad a = 3.0398,$<br>$b = 0.4611, \quad D = 22.5349$
9	Poly.	IV	uniaxial<br>$\bar{u}_1/L = 0.2,\ \bar{u}_2 = 0$	$C_{10} = 0.1656, \quad C_{20} = 2.4958,$<br>$C_{02} = 1.3797, \quad C_{04} = 0.2843,$<br>$C_{42} = 0.1132, \quad D_1 = 0.1324,$<br>$D_2 = 0.1212, \quad D_4 = 0.0306$
10	Ogden	III	uniaxial<br>$\bar{u}_1/L = 0.3,\ \bar{u}_2 = 0$	$\mu_1 = 88.1378, \quad \mu_2 = 91.6428,$<br>$\mu_3 = 82.8531, \quad \mu_4 = 73.1291,$<br>$\mu_5 = 11.3618, \quad \mu_6 = 98.2382,$<br>$\alpha_1 = 13.4329, \quad \alpha_2 = 17.0617,$<br>$\alpha_3 = 16.3020, \quad \alpha_4 = 7.9690,$<br>$\alpha_5 = 8.9464, \quad \alpha_6 = 2.1765,$<br>$D_1 = 62.4539, \quad D_2 = 72.2618,$<br>$D_3 = 73.2496, \quad D_4 = 13.8722$

Note: The parameters μ, C, and D are in MPa.

Table S6. Mean neighbor-to-random ratios based on the symmetric response distances over the 400 query embeddings.

Response signature	Same-material exclusion	Same-class exclusion
Energy gradient R^g	0.150	0.363
Energy profile R^ψ	0.102	0.327

References

1. RS Rivlin, DW Saunders, Large elastic deformations of isotropic materials vii. experiments on the deformation of rubber. *Philos. Transactions Royal Soc. London, Ser. A: Math. Phys. Sci.* **243**, 251–288 (1951).
2. S Hartmann, Numerical studies on the identification of the material parameters of rivlin's hyperelasticity using tension-torsion tests. *Acta Mech.* **148**, 129–155 (2001).
3. RW Ogden, Large deformation isotropic elasticity–on the correlation of theory and experiment for incompressible rubberlike solids. *Proc. Royal Soc. London. A. Math. Phys. Sci.* **326**, 565–584 (1972).
4. B Kim, et al., A comparison among Neo-Hookean model, Mooney-Rivlin model, and Ogden model for chloroprene rubber. *Int. J. Precis. Eng. Manuf.* **13**, 759–764 (2012).
5. S Budday, et al., Mechanical characterization of human brain tissue. *Acta Biomater.* **48**, 319–340 (2017).
6. AN Gent, A new constitutive relation for rubber. *Rubber Chem. Technol.* **69**, 59–61 (1996).
7. E Pucci, G Saccomandi, A note on the Gent model for rubber-like materials. *Rubber Chem. Technol.* **75**, 839–852 (2002).
8. H Khajehsaeid, J Arghavani, R Naghdabadi, A hyperelastic constitutive model for rubber-like materials. *Eur. J. Mech.* **38**, 144–151 (2013).
9. HG Kilian, H Enderle, K Unseld, The use of the van der Waals model to elucidate universal aspects of structure-property relationships in simply extended dry and swollen rubbers. *Colloid Polym. Sci.* **264**, 866–876 (1986).
10. G Marckmann, E Verron, Comparison of hyperelastic models for rubber-like materials. *Rubber Chem. Technol.* **79**, 835–858 (2006).
11. L Li, CQ Chen, Equilibrium-based convolution neural networks for constitutive modeling of hyperelastic materials. *J. Mech. Phys. Solids* **164**, 104931 (2022).
12. L Li, S Li, H Gao, CQ Chen, ENNStressNet - An unsupervised equilibrium-based neural network for end-to-end stress mapping in elastoplastic solids. *J. Mech. Phys. Solids* **200**, 106117 (2025).
13. P Virtanen, et al., Scipy 1.0: fundamental algorithms for scientific computing in python. *Nat. Methods* **17**, 261–272 (2020).
